# Supplementary material for: Feedforward growth rate control mitigates gene activation burden
Source: Nat Commun. 2022 Nov 17;13:7054. doi: 10.1038/s41467-022-34647-1 (PMC9672102; doi:10.1038/s41467-022-34647-1)
Supplement: Supplementary file 1 — Supplementary Information [file 41467_2022_34647_MOESM1_ESM.pdf]

Supplementary to "Feedforward growth rate control mitigates gene activation burden"

Carlos Barajas[1], Hsin-Ho Huang[1], Jesse Gibson[2], Luis Sandoval[3], and Domitilla Del Vecchio[1,*]

[1] Department of Mechanical Engineering, Massachusetts Institute of Technology, Cambridge, Massachusetts

[2] Department of Bioengineering, Stanford University, Palo Alto, California

[3] Department of Chemical Engineering, Massachusetts Institute of Technology, Cambridge, Massachusetts

* Correspondence: ddv@mit.edu

In the first section of the Supplementary Information we provide supplementary experimental results. Then, we provide a detailed mathematical derivation of the SpoTH actuator model. Finally, we provide the plasmid maps of the constructs used in this study along with DNA sequence of nonstandard parts. We end with supplementary notes.

Additional experimental data

The GFP production rate data as SpoTH is expressed corresponding to Fig. 2 in the main text, is shown in Supplementary Fig. 1. The RFP production rate vs growth rate data for all the CL RBS values tested for the experiment corresponding to Fig. 3 in the main text, is shown in Supplementary Fig. 2. The GFP production rate data as the GOI is activated corresponding to Fig. 3 in the main text, is shown in Supplementary Fig. 3. For the feedforward controller from Fig. 3-a in the main text, we replaced SpoTH with a nonfunctional heterologous protein CJB (cjBlue H197S [1]) and call this the control system. The growth rate and GFP production rate data as RFP is activated for the OL system and the control system is shown in Supplementary Fig. 4. The GFP production rate data as RelA+ and SpoTH are expressed corresponding to Fig. 4 in the main text, is shown in Supplementary Fig. 5. The RFP production rate vs growth rate data for all the CL RBS values tested for the experiment corresponding to Fig. 6 in the main text, is shown in Supplementary Fig. 6. The GFP production rate data as the GOI is activated corresponding to Fig. 6 in the main text, is shown in Supplementary Fig. 7. The fluorescence per-cell and growth rate of the same biological replicates as in Fig. 7 were simultaneously tracked individually in a mono-culture (Supplementary Fig. 8). The growth curves for the mono-cultures and co-cultures corresponding to Fig. 7 in the main text are shown in Supplementary Fig. 9.

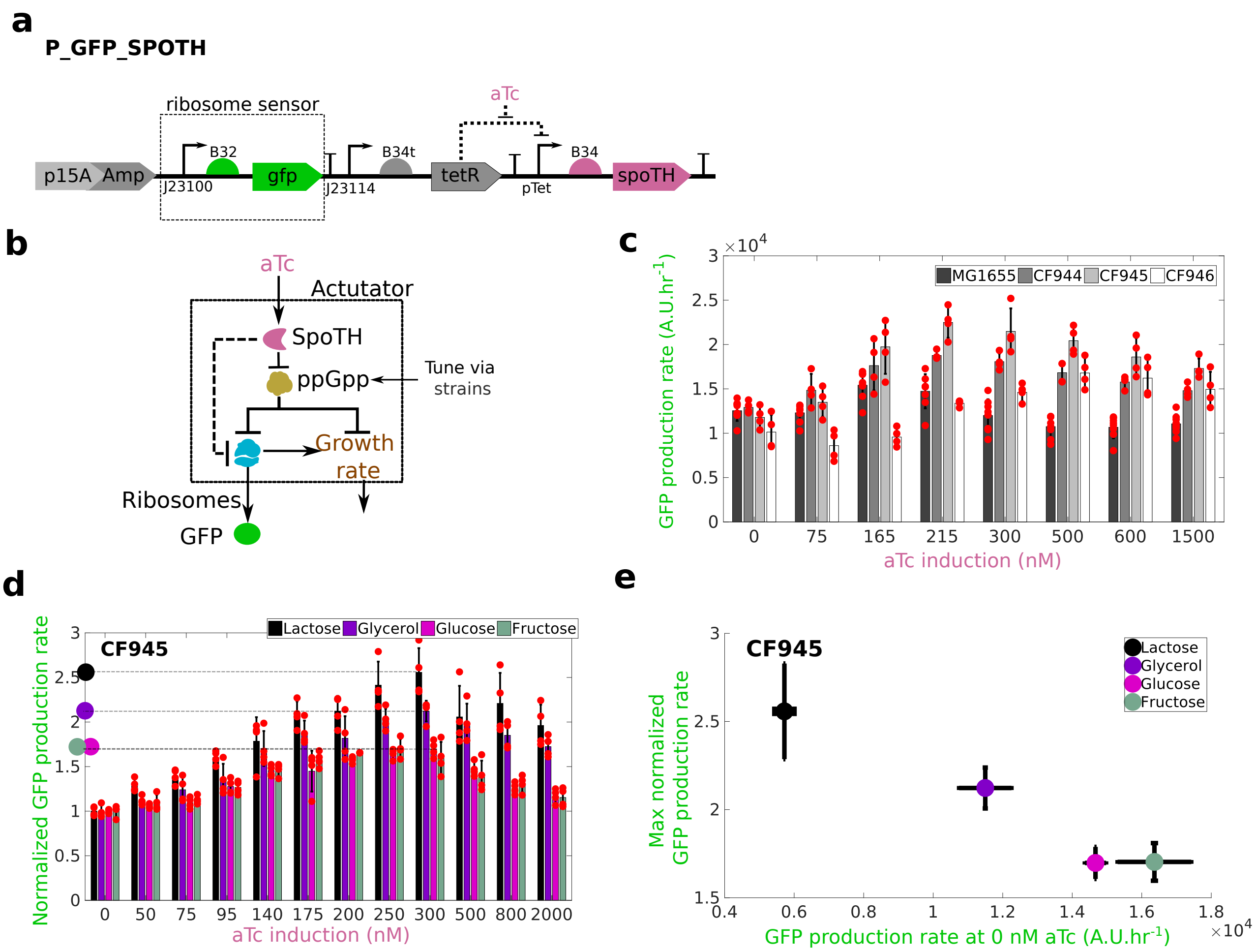

Supplementary Figure. 1: **SpoTH expression increases GFP production rate.** **(a)** The P_GFP_SpoTH plasmid used to express SpoTH via the inducible pTet promoter. Plasmid description, plasmid map, and essential DNA sequences are provided in Supplementary section *Plasmid maps and DNA sequences.* **(b)** Addition of aTc increases SpoTH concentration, which lowers ppGpp concentration and consequently upregulates both free ribosome concentration and growth rate [2]. Here the constitutive GFP production rate serves as a proxy for free ribosomes (Supplementary note 4). **(c)** The GFP production rate while increasing SpoTH in the wild-type MG1655, CF944, CF945, and CF946 strains [2] growing in glycerol as the sole carbon source. **(d)** The GFP production rate normalized by the GFP production rate at aTc = 0 nM, as SpoTH is expressed in CF945 growing in lactose, glycerol, fructose, or glucose as the sole carbon source. The max normalized GFP production rate for each carbon source is marked by open squares. **(e)** The max normalized GFP production rate versus the GFP production rate at aTc = 0 nM for each carbon source. Data are shown as the mean ± one standard deviation (N=4, two biological replicates each with two technical replicates). Individual experimental values are presented as red dots. The complete experimental protocol is provided in the Materials and Methods section.

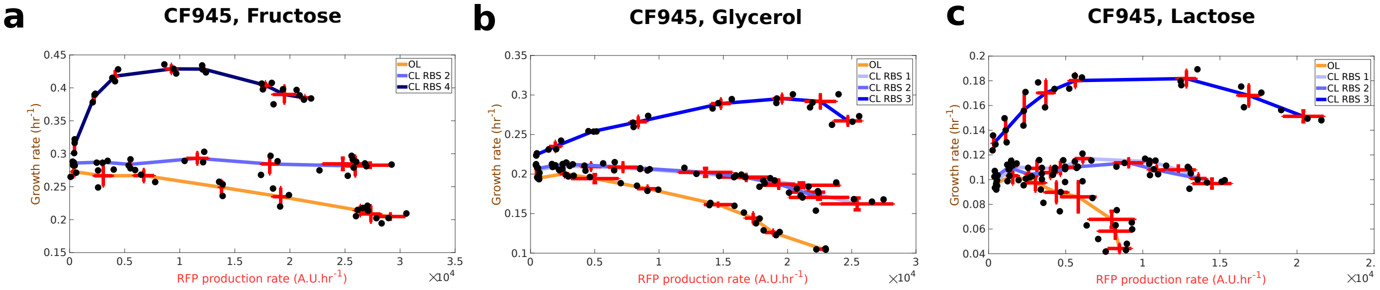

Supplementary Figure. 2: **Feedforward ribosome controller compensates for burden caused by a GOI (RFP) activation.** The unnormalized growth rate versus RFP production rate for all the tested RBS for the CL system corresponding to Fig. 3-e,f,g in the main text. **(a)-(c)** Growth rate versus the RFP production rate for the OL and CL systems, using fructose (a) AHL= [0, 2, 3.5, 7, 12, 35, 50, 100] (nM), glycerol (b) AHL= [0, 1, 2, 3.5, 7, 10, 15, 35] (nM), and lactose (c) AHL= [0, 1, 2, 3, 4, 7, 10, 15] as the carbon source. Data are shown as the mean ± one standard deviation (N=3, three biological replicates). All experiments were performed in the CF945 strain. Individual experimental values are presented as black dots. The complete experimental protocol is provided in the Materials and Methods section. Plasmid description, plasmid map, and essential DNA sequences are provided in Supplementary section *Plasmid maps and DNA sequences*.

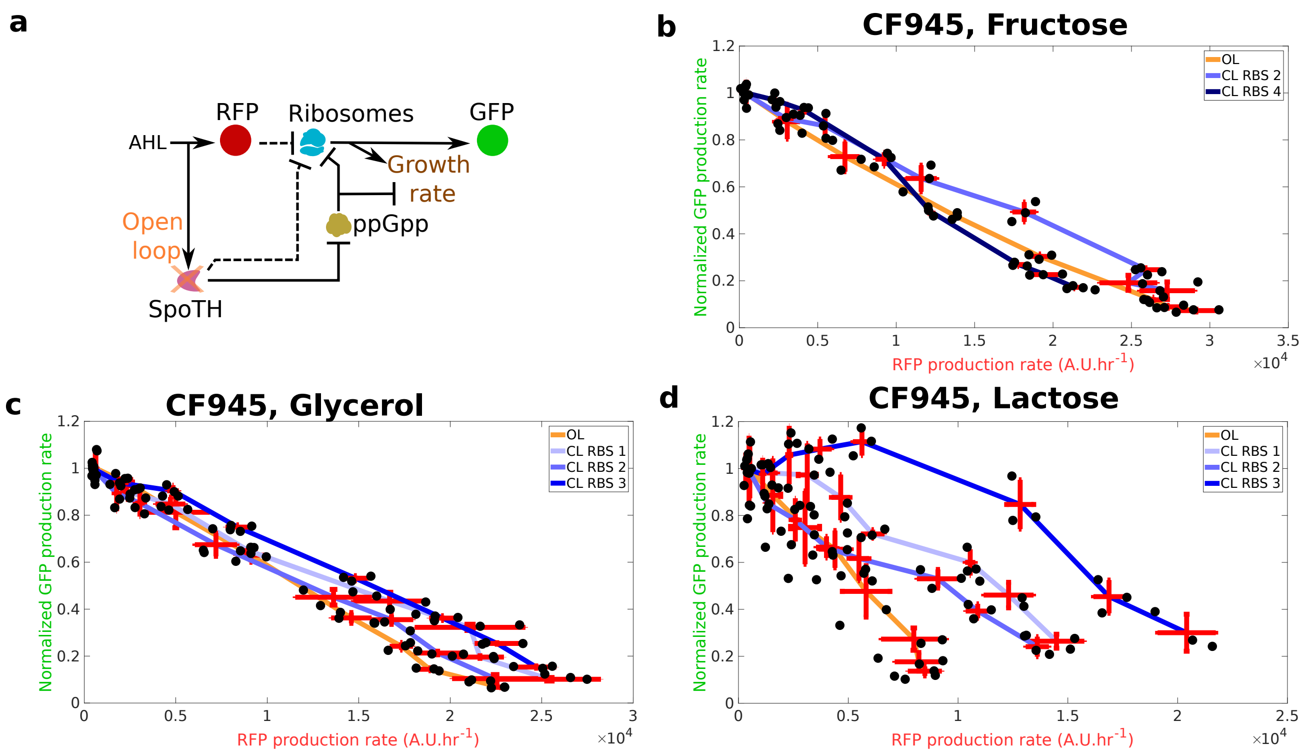

Supplementary Figure. 3: **Feedforward controller compensates for GFP production rate defect caused by a GOI (RFP) activation at low growth rates. (a)** Diagram depicting the effect of expressing RFP (via AHL) on ribosomes and growth rate for the open loop (OL) or closed loop (CL) systems. In the OL system, SpoTH is not present, so there is only the upper path from AHL to ribosomes. In the CL system, AHL also activates SpoTH expression and hence upregulates ribosome concentration and growth rate. Dashed edges represent sequestration of free ribosomes by a protein's mRNA. Here the constitutive GFP production rate serves as a proxy for free ribosomes (Supplementary Note 4). **(b-d)** The GFP production rate normalized by the GFP production rate at 0 nM AHL versus the RFP production rate for the control, OL, and CL systems, using fructose (b) AHL= [0, 2, 3.5, 7, 12, 35, 50, 100] (nM), glycerol (c) AHL= [0, 1, 2, 3.5, 7, 10, 15, 35] (nM), and lactose (d) AHL= [0, 1, 2, 3, 4, 7, 10, 15] as the carbon source. Data are shown as the mean ± one standard deviation (N=3, three biological replicates). All experiments were performed in the CF945 strain. Individual experimental values are presented as black dots. The complete experimental protocol is provided in the Materials and Methods section. Plasmid description, plasmid map, and essential DNA sequences are provided in Supplementary section *Plasmid maps and DNA sequences*.

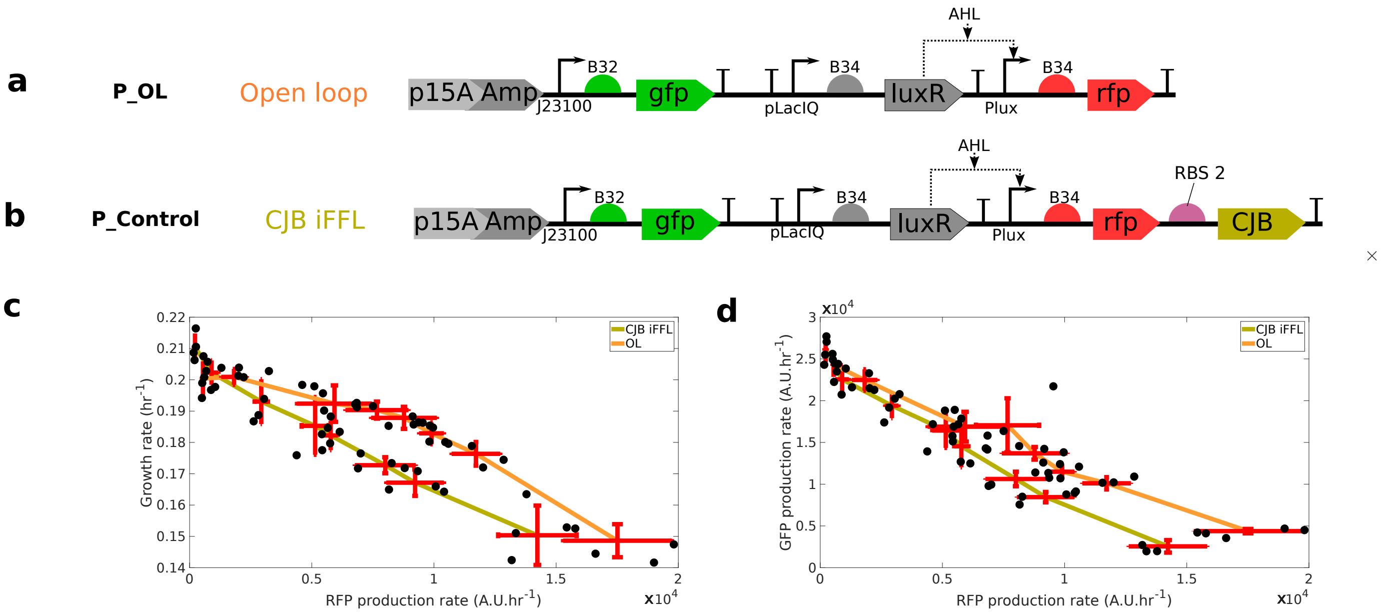

Supplementary Figure. 4: **Replacing SpoTH with CJB in Feedforward controller creates more burden on growth rate and GFP production rate than the OL system.** **(a)** OL system's genetic construct (P_OL) used to express RFP using the AHL (TX input) inducible Plux promoter **(b)** The control genetic construct (P_Control) used to simultaneously express RFP and CJB. The CJB protein is a nonfunctional heterologous protein. **(c)/(d)** Growth rate/GFP production rate versus the RFP production rate for the control and OL systems. Data are shown as the mean ± one standard deviation (N=4, two biological replicates each with two technical replicates). Individual experimental values are presented as a black dots. All experiments were performed in the CF945 strain in media with glycerol as the sole carbon source. The complete experimental protocol is provided in the Materials and Methods section.

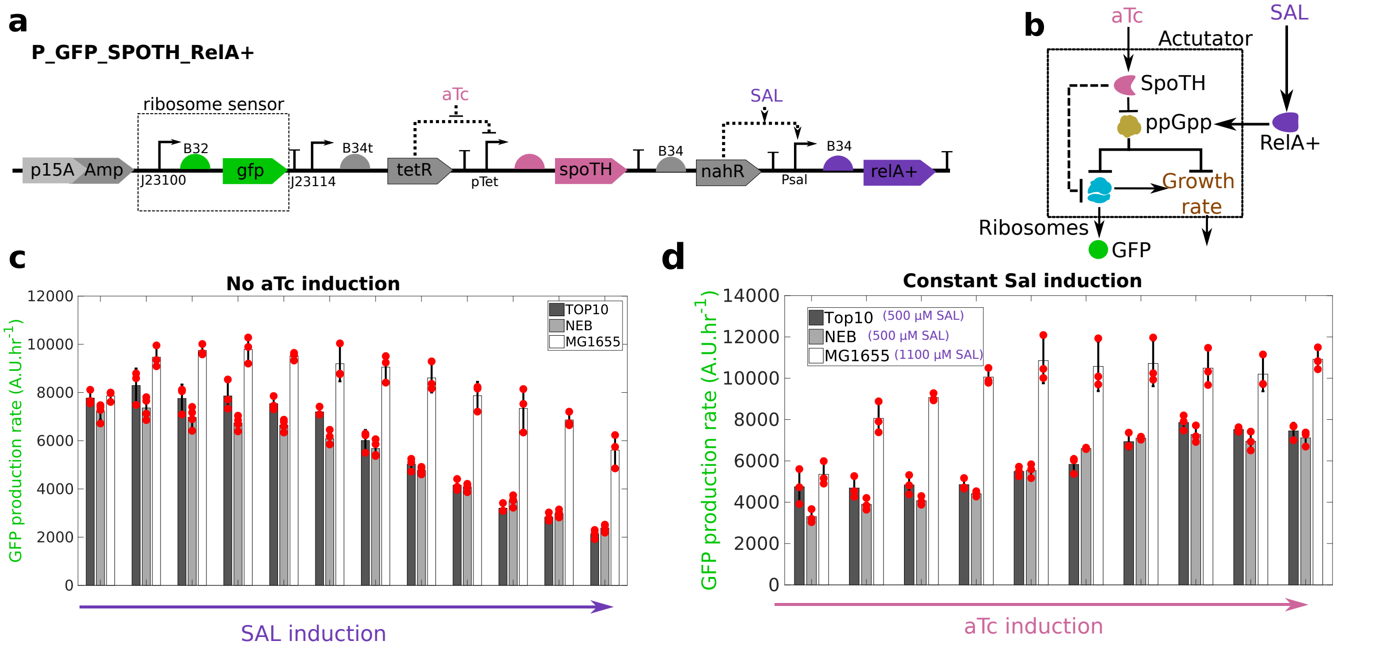

Supplementary Figure. 5: **Expression of RelA+ allows to transport the SpoTH actuator to common laboratory strains.** **(a)** The P_GFP_SpoTH_RelA+ plasmid used to express SpoTH via the inducible pTet promoter and RelA+ via the inducible Psal promoter. Plasmid description, plasmid map, and essential DNA sequences are provided in Supplementary section *Plasmid maps and DNA sequences*. **(b)** Addition of SAL increases RelA+ concentration and thus upregulates ppGpp concentration [3]. Addition of aTc increases SpoTH concentration, which lowers ppGpp concentration and consequently upregulates both free ribosome concentration and growth rate [2]. Here the constitutive GFP production rate serves as a proxy for free ribosomes (Supplementary Note 4). **(c)** GFP production rate versus RelA+ induction (SAL) in the TOP10, NEB, and wild-type MG1655, strains growing in glycerol as the sole carbon source. **(d)** GFP production rate versus SpoTH expression (atc) for a fixed RelA+ expression in TOP10, NEB, and wild-type MG1655 strains growing in glycerol as the sole carbon source. Data are shown as the mean ± one standard deviation (N=3, three biological replicates). All experiments were performed with glycerol as the sole carbon source. Individual experimental values are presented as a red dots. The complete experimental protocol is provided in the Materials and Methods section.

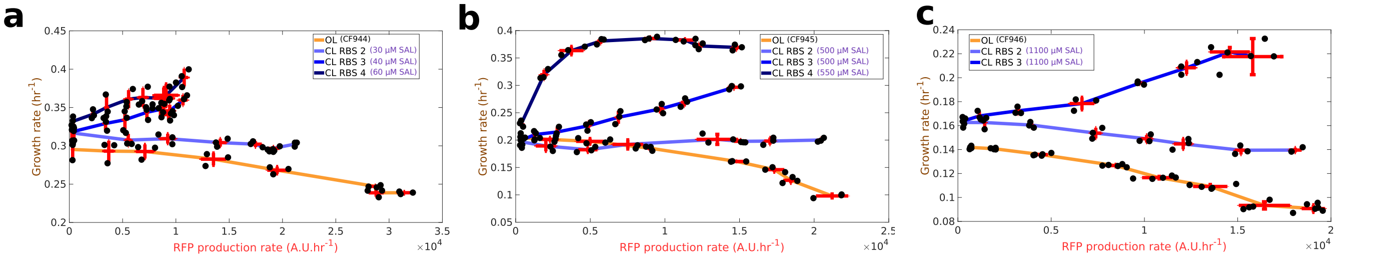

Supplementary Figure. 6: **Feedforward ribosome controller compensates for burden caused by a GOI (RFP) activation in common bacterial strain.** The unnormalized growth rate versus RFP production rate for all the tested RBS for the CL system corresponding to Fig. 5-c,d,e in the main text. **(a)-(c)** Growth rate versus the RFP production rate for the OL in CF944 (a) AHL = [0, 2, 3.5, 7, 12.5, 35, 50, 100] (nM) , CF945 (b) AHL = [0, 1, 2, 3.5, 7, 10, 15, 35] (nM), and CF946 (c) AHL = [0, 0 1 2.5 5 7.5 10 20 30] (nM), and CL systems in TOP10. For the CL system, RelA+ expression is set to match the growth rate of the OL strain. All experiments were performed with glycerol as the sole carbon source. Data are shown as the mean ± one standard deviation (N=3, three biological replicates). All experiments were performed in the CF945 strain. Individual experimental values are presented as black dots. The complete experimental protocol is provided in the Materials and Methods section. Plasmid description, plasmid map, and essential DNA sequences are provided in Supplementary section *Plasmid maps and DNA sequences*.

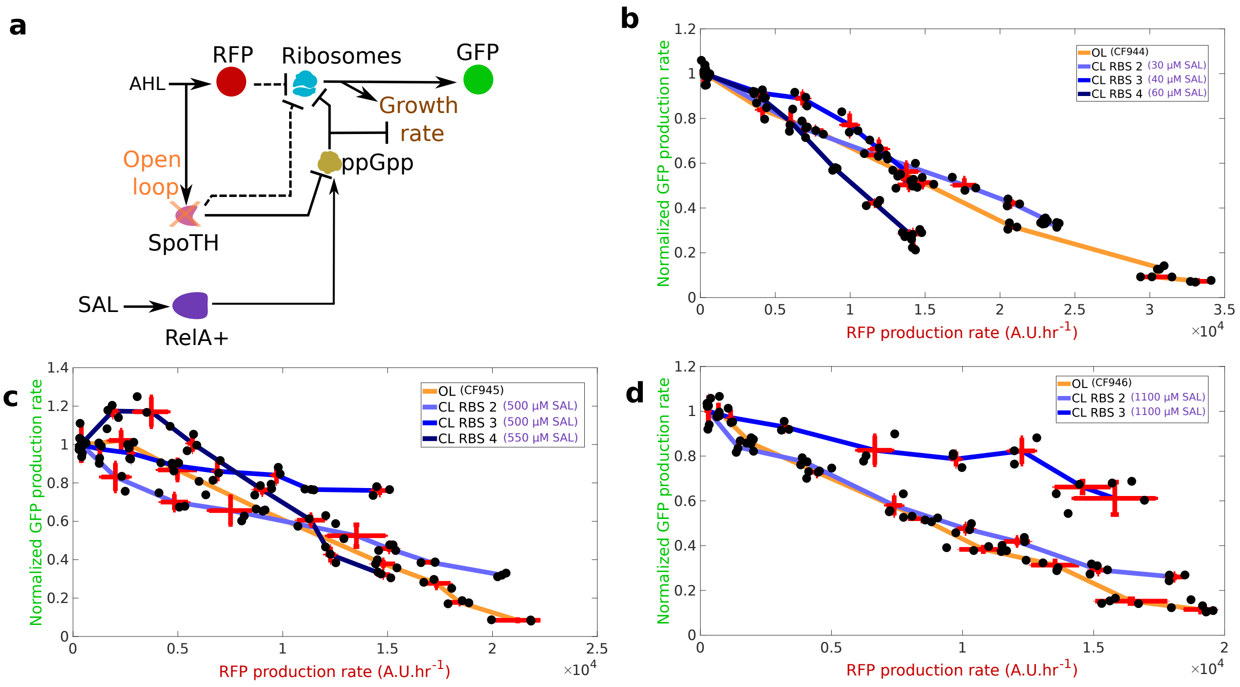

Supplementary Figure. 7: **Feedforward controller compensates for GFP production defects caused by activation of RFP in TOP10 strain at low growth rates.** **(a)** Diagram depicting the effect of activating RFP (AHL input) on ribosomes and growth rate for the open loop (OL) or closed loop (CL) systems. In the OL system, SpoTH is not present, so there is only the upper path from AHL to growth rate. In the CL system, the TX input also activates SpoTH production and hence upregulates ribosome concentration and growth rate. Dashed edges represent sequestration of free ribosomes by a protein mRNA. RelA+ activation via SAL sets the basal level of ppGpp and thus the setpoint growth rate [3]. Here the constitutive GFP production rate serves as a proxy for free ribosomes (Supplementary Note 4). **(c-e)** GFP production rate normalized by the GFP production rate at 0 nM AHL versus the RFP production rate for the OL in CF944 (b) AHL = [0, 2, 3.5, 7, 12.5, 35, 50, 100] (nM) , CF945 (c) AHL = [0, 1, 2, 3.5, 7, 10, 15, 35] (nM), and CF946 (d) AHL = [0, 0 1 2.5 5 7.5 10 20 30] (nM), and CL systems in TOP10. For the CL system, RelA+ expression is set to match the growth rate of the OL strain. Data are shown as the mean ± one standard deviation (N=3, three biological replicates). All experiments were performed with glycerol as the sole carbon source. Individual experimental values are presented as black dots. The complete experimental protocol is provided in the Materials and Methods section. Plasmid description, plasmid map, and essential DNA sequences are provided in Supplementary section *Plasmid maps and DNA sequences*.

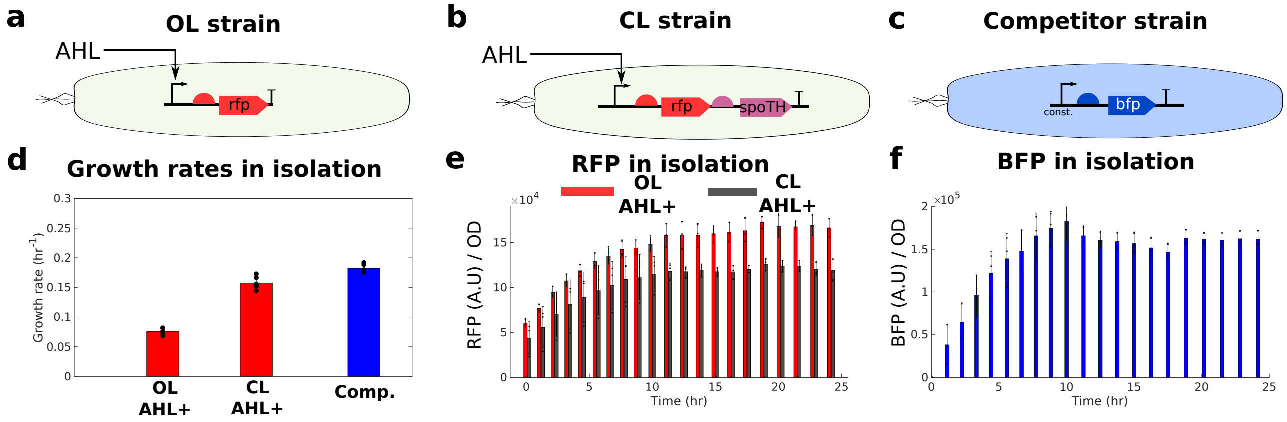

Supplementary Figure. 8: **RFP and BFP expressed persistently in isolation.** **(a)** The OL strain consists of P_OL in CF945. **(b)** The iFFL strain consists of P_IFFL_1 in CF945. **(c)** The competitor strain has P_BFP in TOP10. **(d)** The growth rates for each strain with AHL (AHL+, 27.5 nM) grown in isolation (mono-culture). The growth rates were calculated by averaging the growth rates of the last two batches in Supplementary Fig. 9-a,b for each biological replicate. **(e)** The temporal response of mean RFP per OD values for the OL and CL strain with AHL (27.5 nM). **(f)** The temporal response of mean BFP per OD values for the competitor strain. Data are shown as the mean ± one standard deviation (N=3, three biological replicates). Individual experimental values are presented as a black dots. All experiments were performed in media with glycerol as the sole carbon source. Individual experimental values are presented as black dots. The complete experimental protocol is provided in the Materials and Methods section. Plasmid description, plasmid map, and essential DNA sequences are provided in Supplementary section *Plasmid maps and DNA sequences.*

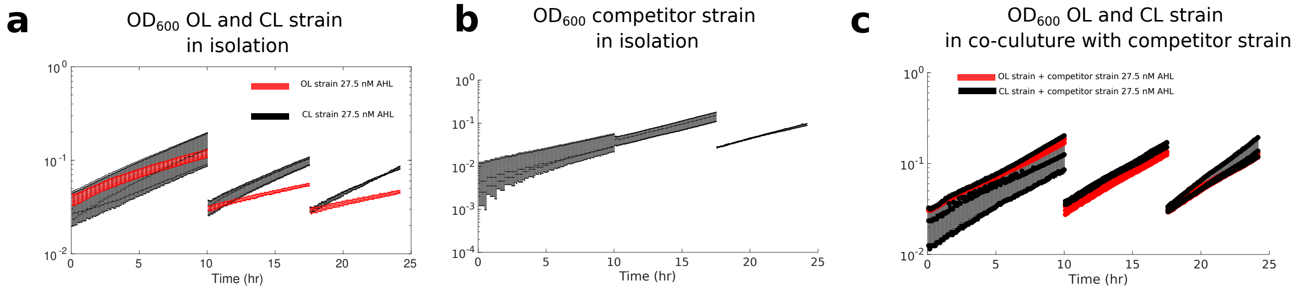

Supplementary Figure. 9: **Co-culture experiment growth curves.** **(a)** The optical density versus time for the OL and CL strain growing in mono-culture with AHL induction. **(b)** The optical density versus time for competitor strain growing in mono-culture. **(c)** The optical density versus time for the co-cultures of OL and competitor strain and the CL and competitor strain growing in co-culture with AHL induction. Data are shown as the mean ± one standard deviation (N=3, three biological replicates). Individual experimental values are presented as dots. All experiments were performed in media with glycerol as the sole carbon source. Individual experimental values are presented as black dots. The complete experimental protocol is provided in the Materials and Methods section. Plasmid description, plasmid map, and essential DNA sequences are provided in Supplementary section *Plasmid maps and DNA sequences.*

Derivation of the SpoTH actuator mathematical model

Following the deterministic modeling framework in [4] and previously applied in [5, 6], we derive a model of the SpoTH actuator. We model SpoTH mRNA being translated by ribosomes to produce the SpoTH protein, which catalyzes the hydrolysis of ppGpp. We model ppGpp inhibiting ribosome production and thus modifying the total ribosomal budget. The resulting dimensional model contains many free parameters, by nondimensionalizing the equations, we can reduce our governing equation

to contain only two dimensionless parameters. Finally, we modify the equations to account for the expression of a heterologous protein.

This modeling framework is not meant to be comprehensive, but rather contain sufficient fidelity to make mathematically precise the physical processes discussed in the main text. The mathematical model is meant to complement the physical intuition provided in the main text used to explain the experimental data.

SpoTH expression and ppGpp hydrolysis and synthesis

We model SpoTH mRNA ($\mathrm{m_s}$) binding to free ribosomes (R) to produce the translation initiation complex $\mathrm{c_s}$, which is then translated to produce the SpoTH protein S with elongation rate constant κ_s. The mRNA decays with rate constants δ_s and the protein dilutes with rate constant γ_s. The corresponding chemical reactions are:

$$\emptyset \xrightarrow{\alpha_\mathrm{s}} \mathrm{m_s}, \quad \mathrm{m_s} \xrightarrow{\delta_\mathrm{s}} \emptyset, \quad \mathrm{m_s} + \mathrm{R} \underset{\mathrm{d_s}}{\overset{\mathrm{a_s}}{\rightleftharpoons}} \mathrm{c_s} \xrightarrow{\kappa_\mathrm{s}} \mathrm{m_s} + \mathrm{R} + \mathrm{P_s}, \quad \mathrm{S} \xrightarrow{\gamma_\mathrm{s}} \emptyset, \tag{1}$$

where α_s is the production rate constant of the mRNA, a_s and d_s are the association and dissociation rate constant, respectively, between ribosomes and mRNA. Levering reaction rate equations, consequently, the concentration of each species satisfies:

$$\begin{aligned}\frac{dm_s}{dt} &= \alpha_s - a_s R m_s + (d_s + \kappa_s) c_s - \delta_s m_s,\\ \frac{dc_s}{dt} &= a_s R m_s - (d_s + \kappa_s) c_s \\ \frac{dS}{dt} &= \kappa_s c_s - \gamma_s S.\end{aligned} \tag{2}$$

The steady state of (2) is given by

$$m_s = \frac{\alpha_s}{\delta_s}, \quad c_s = \frac{m_s}{K_s} R, \quad S = \frac{\kappa_s}{\gamma_s} c_s, \tag{3}$$

where $K_s = \frac{d_s + \kappa_s}{a_s}$. From (3), the concentration of SpoTH S is proportional to c_s (the number of ribosomes translating SpoTH mRNA).

We model RelA (A) and endogenous SpoT ($\mathrm{S_0}$) catalyzing the synthesis of ppGpp (G) (as in Fig. 1 in the main text) from GTP/GDP ($\mathrm{G_P}$), with rates s_1 and s_2, respectively. We model endogenous SpoT and SpoTH catalyzing the hydrolysis of ppGpp to GTP/GDP ($\mathrm{G_P}$) with rates h_1 and h_2, respectively. For simplicity, we model these processes using a *one-step reaction model* [4], that is,

$$\mathrm{A} + \mathrm{G_P} \xrightarrow{\mathrm{s_1}} \mathrm{G} + \mathrm{A}, \quad \mathrm{S_0} + \mathrm{G_P} \xrightarrow{\mathrm{s_2}} \mathrm{G} + \mathrm{S_0}, \quad \mathrm{S_0} + \mathrm{G} \xrightarrow{\mathrm{h_1}} \mathrm{G_P} + \mathrm{S_0}, \quad \mathrm{S} + \mathrm{G} \xrightarrow{\mathrm{h_2}} \mathrm{G_P} + \mathrm{S}. \tag{4}$$

The concentration of ppGpp satisfies:

$$\frac{dG}{dt} = \alpha_G - \gamma_{G_0} G - h_2 G S, \tag{5}$$

where $\alpha_G = s_1 A G_P + s_2 S_0 G_P$ is the effective production rate and $\gamma_{G_0} = h_1 S_0$ is the basal decay rate. The steady state of (5) is given by

$$G = \frac{G_0}{1 + S/K_{gs}}, \tag{6}$$

where $K_{gs} = \gamma_{G_0}/h_2$ and $G_0 = \alpha_G/\gamma_{G_0}$. The quantity G_0 corresponds to the basal ppGpp in the cell ($G(S = 0) = G_0$). The quantity G_0 is varied experimentally via chromosolmal mutation (e.g., *spot203*), media carbon source, and RelA+ expression (Fig. 2 and Fig. 4 in the main text).

Actuating the ribosomal budget in the cell

The concentration of total ribosomes in the cell (R_T), known as the ribosomal budget [5], is composed of free ribosomes, the portion of ribosomes translating endogenous mRNAs (c_e), and the portion of ribosomes translating SpoTH mRNA, that is,

$$R_T = R + c_s + c_e. \tag{7}$$

The total ribosome concentration obeys

$$\frac{dR_T}{dt} = \alpha_r - \gamma_r R_T, \tag{8}$$

where α_r is the ribosome production rate, γ_r is the ribosome decay rate. If α_r and γ_r are assumed time invariant and $R_T(0) = \frac{\alpha_r}{\gamma_r}$, then $R_T(t) = \frac{\alpha_r}{\gamma_r}, \forall t \geq 0$. A temporally constant ribosome budget is consistent with the modeling framework of [5, 6, 7]. However, in this work α_r is not constant and is the term that links ribosome and ppGpp concentration.

Ribosome production (α_r) is set by rRNA production (this the rate-limiting step) [8, 9]. rRNA is expressed from seven rRNA operons (*rrn* operons) [10] each driven by two tandem promoters P1 and P2. Most rRNA transcription arises from the P1 promoter and it is the main “knob” for ribosome tuning except at very low growth rates where P2 regulation dominates [11]. During balanced exponential growth, ppGpp is the primary regulator of rRNA[8, 12, 13] by destabilizing the open RNAP-P1 promoter complex [14, 15]. Therefore, there is an inverse relationship between basal ppGpp levels and rRNA transcription [2, 16, 17, 18]. A simple model to capture this process (previously used in [19]), is given by

$$\alpha_r = \frac{\alpha_r^*}{1 + (G/K_G)^2}, \tag{9}$$

where K_G is the effective dissociation constant between ppGpp and the P1 promoter and α_r^* is the ribosome production rate in the absence of ppGpp ($\alpha_r(G = 0)$). The hill coefficient of 2 in (9) is consistent with the findings of [10]. Taking the steady state of (8) and levering (3), (6), and (9), we have that

$$R_T = \frac{R_T^*}{1 + (G/K_G)^2} = \frac{R_T^*}{1 + (\frac{G_0}{K_G}(\frac{1}{1+c_s\frac{\kappa_s}{K_{gs}\gamma_s}}))^2},$$

where $R_T^* = \alpha_r^*/\gamma_r$.

Rewriting (7), we have at steady state that

$$\frac{R_T^*}{1 + (\frac{G_0}{K_G}(\frac{1}{1+c_s\frac{\kappa_s}{K_{gs}\gamma_s}}))^2} = R + c_s + c_e, \tag{10}$$

making explicit the relationship between basal ppGpp concentration (G_0) and the total ribosomal budget and how increasing SpoTH expression (increasing c_s) both increases the total ribosomal budget (LHS) but also sequesters ribosomes (RHS) via translation demand.

By adding and subtracting $\frac{1}{1+(G_0/K_G)^2}$ to the LHS of (7) and dividing both sides by R_T^*, we can rewrite of (7) as

$$\frac{1}{1 + (G_0/K_G)^2} + \Big[\frac{1}{1 + (\frac{G_0}{K_G}(\frac{1}{1+c_s\frac{\kappa_s}{K_{gs}\gamma_s}}))^2} - \frac{1}{1 + (G_0/K_G)^2}\Big] = \frac{R + c_e}{R_T^*} + \frac{c_s}{R_T^*}. \tag{11}$$

Modeling c_e requires knowing the concentration of the mRNA-ribosome complex for every mRNA expressed by an endogenous gene and thus it is difficult to write an explicit expression. Instead of modeling c_e explicitly, we keep it as a general function of R. We assume that the concentration of $c_e(R)$ monotonically increases with free ribosomes, that is, $\frac{dc_e}{dR} > 0$. This assumptions is reasonable since a steady state complex concentration is proportional to the concentrations of the reacting species [20] (also (3)). Next, we define the following variable that serves as a proxy for free ribosomes:

$$z(R) = R + c_e(R).$$

From the assumption that $\frac{dc_e}{dR} > 0$, it implies that the map $z(R)$ is one-one and thus for every value of R there is a unique corresponding value of z and that an increase/decrease in R corresponds to an increase/decrease in z. Furthermore, we have that $z(0) = 0$ since no the complex c_e cannot be formed without the reactant species R. Therefore, from here on, we refer to $z(R)$ as the *modified free ribosome concentration.*

Example: if we assume that $c_e(R)$ had a form similar to that as c_s as given by (3), then, for q different endogenous genes expressing mRNA, $c_e(R) = \sum_{i=1}^{q} \frac{m_{e,i}}{K_{e,i}} R$, where for gene i, $m_{e,i}$ is the endogenous mRNA concentration and $K_{e,i}$ is the effective dissociation constant of endogenous mRNA with ribosomes. In this cases, $c_e(R)$ satisfies all of our assumptions and furthermore, $z(R)$ is simply proportional to R. However, in all of our analysis we do not explicitly specify $c_e(R)$.

We denote $\bar{z} = \frac{z}{R_T^*}$ and $\bar{c}_s = \frac{c_s}{R_T^*}$ and express (11) in dimensionless form as

$$\bar{z}_0 + (1 - \bar{z}_0) f(\bar{c}_s/\epsilon, \bar{z}_0) = \bar{z} + \bar{c}_s, \tag{12}$$

where

$$\theta_G = G_0/K_G, \quad \bar{z}_0(\theta_G) = \frac{1}{1+\theta_G^2}, \quad \epsilon = \frac{K_{gs}}{R_T^*}\frac{\gamma_s}{\kappa_s}, \quad f(\bar{c}_s/\epsilon, \bar{z}_0) = \frac{(\bar{c}_s/\epsilon + 1)^2 - 1}{(\bar{c}_s/\epsilon + 1)^2 - 1 + 1/\bar{z}_0}. \tag{13}$$

The dimensionless parameter θ_G is a measure of the basal ppGpp in the cell, $\bar{z}_0$ is the dimensionless modified free ribosome concentration when no SpoTH is expressed ($\bar{c}_s = 0$) and we refer to this quantity as the *nominal modified ribosome level*, ϵ is a measure of the ribosomal cost to express sufficient SpoTH to actuate (catalyze the hydrolysis of a sufficient amount of ppGpp). A small ϵ implies that a small $\bar{c}_s$ is needed to saturate the f term. Also notice that there is a monotonically decreasing relationship between the basal ppGpp θ_G and the nominal modified ribosome level. Finally, a key parameter to determine the qualitative behavior of (12) is given by:

$$\delta(\bar{z}_0, \epsilon) := \left.\frac{d\bar{z}}{d\bar{c}_s}\right|_{\bar{c}_s=0} = 2\frac{\theta_G^2}{(1+\theta_G^2)^2\epsilon} - 1 = \frac{2\bar{z}_0(1-\bar{z}_0)}{\epsilon} - 1, \tag{14}$$

where $\delta \in (-1, \infty)$. By definition and our assumption that $\frac{dc_e}{dR} > 0$, if $\delta > 0$, it implies that ribosome levels increase as a small amount of exogenous SpoT is expressed.

Appending the model with the expression of an additional heterologous protein

We model the mRNA of a heterologous protein ($\mathrm{m_y}$) binding to free ribosomes (R) to produce the translation initiation complex $\mathrm{c_y}$, which is then translated to produce the protein y with elongation rate constant κ_y. The mRNA decays with rate constants δ_y and the protein dilutes with rate constant γ_y. The corresponding chemical reactions are:

$$\emptyset \xrightarrow{\alpha_y} \mathrm{m_y}, \quad \mathrm{m_y} \xrightarrow{\delta_y} \emptyset, \quad \mathrm{m_y} + \mathrm{R} \underset{\mathrm{d_y}}{\overset{\mathrm{a_y}}{\rightleftharpoons}} \mathrm{c_y} \xrightarrow{\kappa_y} \mathrm{m_y} + \mathrm{R} + \mathrm{P_y}, \quad \mathrm{y} \xrightarrow{\gamma_y} \emptyset, \tag{15}$$

where α_y is the production rate constant of the mRNA, a_y and d_y are the association and dissociation rate constant, respectively, between ribosomes and mRNA. The concentration of each species satisfies:

$$\begin{aligned}\frac{dm_y}{dt} &= \alpha_y - a_y R m_y + (d_y + \kappa_y) c_y - \delta_y m_y,\\ \frac{dc_y}{dt} &= a_y R m_y - (d_y + \kappa_y) c_y \\ \frac{dy}{dt} &= \kappa_y c_y - \gamma_y P_y.\end{aligned} \tag{16}$$

The steady state of (16) is given by

$$m_y = \frac{\alpha_y}{\delta_y}, \quad c_y = \frac{m_y}{K_y} R, \quad y = \frac{\kappa_y}{\gamma_y} c_y, \tag{17}$$

where $K_y = \frac{d_y + \kappa_y}{a_y}$. We modify the total ribosome equation (7) to include the ribosomes sequestered by the y mRNA, and it reads

$$R_T = R + c_s + c_e + c_y.$$

Defining $\bar{c}_y = c_y / R_T^*$, the total ribosome concentration in dimensionless from as in (12), is given by

$$\bar{z}_0 + (1 - \bar{z}_0) f(\bar{c}_s/\epsilon, \bar{z}_0) = \bar{z} + \bar{c}_s + \bar{c}_y. \tag{18}$$

If y and SpoTH are under the same promoter, that is $m_y = m_s$, then from (3) and (17) we have that at steady state

$$\bar{c}_s = \gamma \bar{c}_y,$$

where $\gamma = K_y/K_s$ is the SpoTH RBS strength relative to the y RBS strength. We refer to the configuration when SpoTH and y are under the same promoter ($\bar{c}_s = \gamma \bar{c}_y$), as the closed loop and the case when y is expressed in isolation ($\bar{c}_s = 0$ for all $\bar{c}_y$), as the open loop. The qualitative behavior of (18) for the open loop and closed loop is shown in Supplementary Fig. 10. For the close loop, we have can express the initial sensitivity of free ribosome as y is expressed as

$$\left.\frac{d\bar{z}}{d\bar{c}_y}\right|_{\bar{c}_y = 0} = \gamma\delta - 1. \tag{19}$$

Thus, we can make the slope zero (free ribosomes are initially not sensitive to the expression of y) if we choose the SpoTH RBS strength (relative to the y RBS strength) as

$$\gamma = 1/\delta(\bar{z}_0, \epsilon). \tag{20}$$

In Supplementary Fig. 10 we observe that as $\gamma\delta \to -1$, the closed loop performs worst than the open loop (for a given $\bar{c}_y$, the corresponding value of $\bar{z}$ is lower) and the performance gets worst for larger values of γ.

Plasmid maps and DNA sequences

The plasmids used in this study and their description are provided in Table 1. The corresponding plasmid maps are shown in Supplementary Fig. 11. The essential DNA sequences are provided in Table 2. The full plasmid DNA sequences and primers is included in the Supplementary Data.

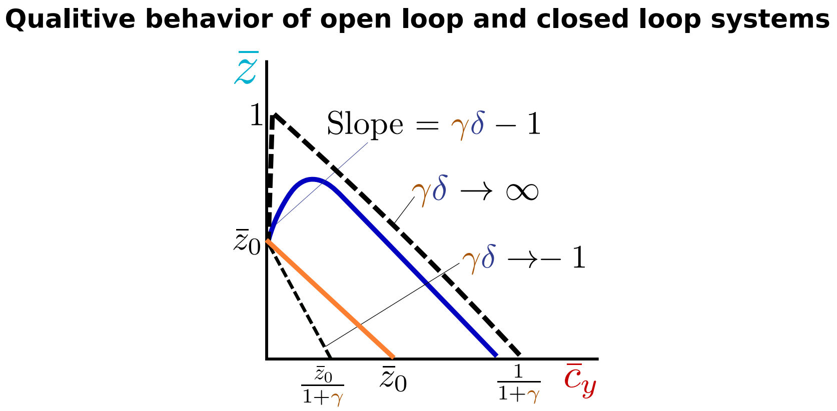

Supplementary Figure. 10: **The qualitative behavior coexpressing** y **and SpoTH under the same promoter.** The qualitative behavior of (18) when $\bar{c}_s = \gamma\bar{c}_y$ (blue line). The open loop ($\bar{c}_s = 0$ for all $\bar{c}_y$) is shown in orange. The asymptotic behaviors as $\gamma\delta \to -1$ and $\gamma\delta \to \infty$ are shown in dashed lines.

Plasmid Name	Plasmid map	Comments
P_GFP_SpoTH	Supplementary Fig. 11-a	SpoTH cloned using pHX41 [21], see Supplementary Note 3<br>The rest of the parts are from pHH03C_32 [7]
P_GFP_SpoTH_RFP	Supplementary Fig. 11-b	P_GFP_SpoTH with luxR-Plux-RFP added from MBP-1.0 [5]
P_GFP_SpoTH_dCas9	Supplementary Fig. 11-c	P_GFP_SpoTH_RFP with RFP replaced by dCas9 from pdCas9_OP in [22]
P_OL	Supplementary Fig. 11-d	P_GFP_SpoTH_RFP with TetR-pTet-SpoTH removed
P_IFFL_1	Supplementary Fig. 11-e	P_OL with RBS 1-SpoTH directly downstream of RFP
P_IFFL_2	Supplementary Fig. 11-e	P_OL with RBS 2-SpoTH directly downstream of RFP
P_IFFL_3	Supplementary Fig. 11-e	P_OL with RBS 3-SpoTH directly downstream of RFP
P_IFFL_4	Supplementary Fig. 11-e	P_OL with RBS 4-SpoTH directly downstream of RFP
P_Control	Supplementary Fig. 11-f	P_IFFL_2 but SpoTH replaced by CJB (cjBlue H197S [1])<br>The CJB DNA is codon optimized for *E. coli*
P_weak_RFP	Supplementary Fig. 11-d	P_OL but changed RFP RBS from B34 to RBS weak (MBP-0.006 in [5])
P_weak_RFP_SpoTH_1	Supplementary Fig. 11-e	P_IFFL_1 but replaced RFP RBS from B34 to RBS weak
P_weak_RFP_SpoTH_2	Supplementary Fig. 11-e	P_IFFL_2 but changed RFP RBS from B34 to RBS weak
P_weak_RFP_SpoTH_3	Supplementary Fig. 11-e	P_IFFL_3 but changed RFP RBS from B34 to RBS weak
P_weak_RFP_SpoTH_4	Supplementary Fig. 11-e	P_IFFL_4 but changed RFP RBS from B34 to RBS weak
P_GFP_SpoTH_RelA+	Supplementary Fig. 11-g	P_GFP_SpoTH but with an inducible RelA+ [3] cassette
P_IFFL_SpoTH_RelA_1	Supplementary Fig. 11-h	P_IFFL_1 with an inducible RelA+ cassette
P_IFFL_SpoTH_RelA_2	Supplementary Fig. 11-h	P_IFFL_2 with an inducible RelA+ cassette
P_IFFL_SpoTH_RelA_3	Supplementary Fig. 11-h	P_IFFL_3 with an inducible RelA+ cassette
P_IFFL_SpoTH_RelA_4	Supplementary Fig. 11-h	P_IFFL_4 with an inducible RelA+ cassette
P_BFP	Supplementary Fig. 11-i	inducible RelA+ cassette with constitutive BFP from [23]

Table 1: Description of plasmids used in this study

Part	Sequence (5' to 3')
B32	TACTAGAGTCACACAGGAAAGTACTAG
B0033	ACTAGAGTCACACAGGACTACTAG
B34	TACTAGAGAAAGAGGAGAAATACTAG
B34t	AATTCATTAAAGAGGAGAAAGGTACC
RBS 1	GTTACAGCTTAGCCCGATCCATTT
RBS 2	TGAGCGCCGCCAGGGACCACGC
RBS 3	CTCGACACACCCCCCTATTAAAT
RBS 4	CAAACCTCTACCGTAGGATTCGTCATT

RBS weak	TCGGCCCTATACGACTCTAAACGTCGATG
RBS BFP	TACGCCGATTCTGCCGGGGGGAATTATA
J23100	TTGACGGCTAGCTCAGTCCTAGGTACAGTGCTAGC
J23114	TTTATGGCTAGCTCAGTCCTAGGTACAATGCTAGC
J23104	TTGACAGCTAGCTCAGTCCTAGGTATTGTGCTAGC
pTet	TCCCTATCAGTGATAGAGATTGACATCCCTATCAGTGATAGAGATACTGAGCAC
pLacIQ	TGGTGCAAAACCTTTCGCGGTATGGCATGATAGCGCC
Plux	ACCTGTAGGATCGTACAGGTTTACGCAAGAAAATGGTTTGTTATAGTCGAATAAA
LuxR	ATGAAAAACATAAATGCCGACGACACATACAGAATAATTAATAAAATTAAAGCTTGTAGAAGC<br>AATAATGATATTAATCAATGCTTATCTGATATGACTAAAATGGTACATTGTGAATATTATTTA<br>CTCGCGATCATTTATCCTCATTCTATGGTTAAATCTGATATTTCAATCCTAGATAATTACCCTA<br>AAAAATGGAGGCAATATTATGATGACGCTAATTTAATAAAATATGATCCTATAGTAGATTATT<br>CTAACTCCAATCATTCACCAATTAATTGGAATATATTTGAAAACAATGCTGTAAATAAAAAATC<br>TCCAAATGTAATTAAAGAAGCGAAAACATCAGGTCTTATCACTGGGTTTAGTTTCCCTATTCA<br>TACGGCTAACAATGGCTTCGGAATGCTTAGTTTTGCACATTCAGAAAAAGACAACTATATAGA<br>TAGTTTATTTTTACATGCGTGTATGAACATACCATTAATTGTTCCTTCTCTAGTTGATAATTAT<br>CGAAAAATAAATATAGCAAATAATAAATCAAACAACGATTTAACCAAAAGAGAAAAAGAATGT<br>TTAGCGTGGGCATGCGAAGGAAAAAGCTCTTGGGATATTTCAAAAATATTAGGTTGCAGTGAG<br>CGTACTGTCACTTTCCATTTAACCAATGCGCAAATGAAACTCAATACAACAAACCGCTGCCAAA<br>GTATTTCTAAAGCAATTTTAACAGGAGCAATTGATTGCCCATACTTTAAAAATTAATAA
TetR	ATGTCCAGATTAGATAAAAGTAAAGTGATTAACAGCGCATTAGAGCTGCTTAATGAGGTCGGA<br>ATCGAAGGTTTAACAACCCGTAAACTCGCCCAGAAGCTAGGTGTAGAGCAGCCTACATTGTAT<br>TGGCATGTAAAAAATAAGCGGGCTTTGCTCGACGCCTTAGCCATTGAGATGTTAGATAGGCAC<br>CATACTCACTTTTGCCCTTTAGAAGGGGAAAGCTGGCAAGATTTTTTACGTAATAACGCTAAA<br>AGTTTTAGATGTGCTTTACTAAGTCATCGCGATGGAGCAAAAGTACATTTAGGTACACGGCCT<br>ACAGAAAAACAGTATGAAACTCTCGAAAATCAATTAGCCTTTTTATGCCAACAAGGTTTTTCA<br>CTAGAGAATGCATTATATGCACTCAGCGCTGTGGGGCATTTTACTTTAGGTTGCGTATTGGAA<br>GATCAAGAGCATCAAGTCGCTAAAGAAGAAAGGGAAACACCTACTACTGATAGTATGCCGCCA<br>TTATTACGACAAGCTATCGAATTATTTGATCACCAAGGTGCAGAGCCAGCCTTCTTATTCGGC<br>CTTGAATTGATCATATGCGGATTAGAAAAACAACTTAAATGTGAAAGTGGGTCCT
GFP	ATGCGTAAAGGAGAAGAACTTTTCACTGGAGTTGTCCCAATTCTTGTTGAATTAGATGGTGAT<br>GTTAATGGGCACAAATTTTCTGTCAGTGGAGAGGGTGAAGGTGATGCAACATACGGAAAACTT<br>ACCCTTAAATTTATTTGCACTACTGGAAAACTACCTGTTCCATGGCCAACACTTGTCACTACTT<br>TCGGTTATGGTGTTCAATGCTTTGCGAGATACCCAGATCATATGAAACAGCATGACTTTTTCA<br>AGAGTGCCATGCCCGAAGGTTATGTACAGGAAAGAACTATATTTTTCAAAGATGACGGGAACT<br>ACAAGACACGTGCTGAAGTCAAGTTTGAAGGTGATACCCTTGTTAATAGAATCGAGTTAAAAG<br>GTATTGATTTTAAAGAAGATGGAAACATTCTTGGACACAAATTGGAATACAACTATAACTCAC<br>ACAATGTATACATCATGGCAGACAAACAAAAGAATGGAATCAAAGTTAACTTCAAAATTAGAC<br>ACAACATTGAAGATGGAAGCGTTCAACTAGCAGACCATTATCAACAAAATACTCCAATTGGCG<br>ATGGCCCTGTCCTTTTACCAGACAACCATTACCTGTCCACACAATCTGCCCTTTCGAAAGATCC<br>CAACGAAAAGAGAGACCACATGGTCCTTCTTGAGTTTGTAACAGCTGCTGGGATTACACATGG<br>CATGGATGAACTATACAAATAATAA
RFP	ATGGCTTCCTCCGAAGACGTTATCAAAGAGTTCATGCGTTTCAAAGTTCGTATGGAAGGTTCC<br>GTTAACGGTCACGAGTTCGAAATCGAAGGTGAAGGTGAAGGTCGTCCGTACGAAGGTACCCAG<br>ACCGCTAAACTGAAAGTTACCAAAGGTGGTCCGCTGCCGTTCGCTTGGGACATCCTGTCCCCG<br>CAGTTCCAGTACGGTTCCAAAGCTTACGTTAAACACCCGGCTGACATCCCGGACTACCTGAAA<br>CTGTCCTTCCCGGAAGGTTTCAAATGGGAACGTGTTATGAACTTCGAAGACGGTGGTGTTGTT<br>ACCGTTACCCAGGACTCCTCCCTGCAAGACGGTGAGTTCATCTACAAAGTTAAACTGCGTGGT<br>ACCAACTTCCCGTCCGACGGTCCGGTTATGCAGAAAAAAACCATGGGTTGGGAAGCTTCCACC<br>GAACGTATGTACCCGGAAGACGGTGCTCTGAAAGGTGAAATCAAAATGCGTCTGAAACTGAAA<br>GACGGTGGTCACTACGACGCTGAAGTTAAAACCACCTACATGGCTAAAAAACCGGTTCAGCTG<br>CCGGGTGCTTACAAAACCGACATCAAACTGGACATCACCTCCCACAACGAAGACTACACCATC<br>GTTGAACAGTACGAACGTGCTGAAGGTCGTCACTCCACCGGTGCTTAATAA
BFP	ATGAGCGAACTGATCAAAGAGAACATGCACATGAAGCTGTACATGGAGGGTACCGTGGATAAT<br>CACCACTTTAAGTGTACTTCTGAGGGCGAGGGTAAGCCGTATGAAGGGACTCAAACGATGCGT<br>ATTAAAGTAGTGGAGGGTGGCCCACTGCCGTTTGCTTTCGATATTCTGGCGACGAGCTTTCTG<br>TATGGTAGCAAAACGTTTATAAACCACACTCAGGGCATTCCGGATTTCTTTAAACAAAGCTTT<br>CCGGAAGGTTTTACCTGGGAGCGTGTGACTACGTATGAAGATGGTGGTGTGTTGACTGCTACT<br>CAAGATACTTCACTGCAGGACGGCTGTCTGATCTATAACGTGAAGATTCGTGGCGTGAACTTT<br>ACGAGCAATGGGCCGGTAATGCAAAAAAAAACCCTGGGTTGGGAAGCGTTCACGGAAACTCTG<br>TATCCGGCTGACGGCGGCCTGGAGGGCCGTAACGATATGGCACTGAAGCTGGTTGGTGGCAGC<br>CACCTGATCGCGAATATCAAAACGACTTATCGCTCTAAAAAACCGGCGAAAAATCTGAAGATG<br>CCGGGTGTTTATTATGTTGACTATCGTCTGGAACGCATTAAAGAAGCGAATAATGAAACTTAC<br>GTGGAGCAACACGAGGTTGCAGTGGCGCGCTATTGCGACTTGCCTTCAAAGCTGGGTCACAAA<br>CTGAATTAA
SpoTH	ATGTATCTGTTTGAAAGCCTGAATCAACTGATTCAAACCTACCTGCCGGAAGACCAAATCAAG<br>CGTCTGCGGCAGGCGTATCTCGTTGCACGTGATGCTCACGAGGGGCAAACACGTTCAAGCGGT<br>GAACCCTATATCACGCACCCGGTAGCGGTTGCCTGCATTCTGGCCGAGATGAAACTCGACTAT<br>GAAACGCTGATGGCGGCGCTGCTGCATGACGTGATTGAAGATACTCCCGCCACCTACCAGGAT<br>ATGGAACAGCTTTTTGGTAAAAGCGTCGCCGAGCTGGTAGAGGGGGTGTCGAAACTTGATAAA<br>CTCAAGTTCCGCGATAAGAAAGAGGCGCAGGCCGAAAACTTTCGCAAGATGATTATGGCGATG<br>GTGCAGGATATCCGCGTCATCCTCATCAAACTTGCCGACCGTACCCACAACATGCGCACGCTG<br>GGCTCACTTCGCCCGGACAAACGTCGCCGCATCGCCCGTGAAACTCTCGAAATTTATAGCCCG<br>CTGGCGCACCGTTTAGGTATCCACCACATTAAAACCGAACTCGAAGAGCTGGGTTTTGAGGCG<br>CTGTATCCCAACCGTTATCGCGTAATCAAAGAAGTGGTGAAAGCCGCGCGCCAAAATTCGTCA<br>GTTGCTGAAAAACCTCAAGCGTAATAA

CJB

ATGCATCATCATCATCATCATGCGTCGAAAATCTCAGACAATGTACGTATTAAACTGTACATG
GAAGGCACCGTCAACAACCATCACTTCATGTGTGAAGCGGAAGGTGAAGGCAAGCCGTACGAA
GGAACCCAGATGGAAAACATTAAAGTCACTAAAGGTGGCCCTCTTCCGTTCTCTTTCGACATT
CTCACGCCGAACTGTCAGTATGGGTCAGTGGCAATCACCAAATATACCAGTGGCATCCCGGAC
TACTTTAAGCAGAGCTTTCCTGAGGGATTCACGTGGGAACGCACGACCATCTATGAAGACGGG
GCCTACCTTACAACCCAACAAGAAACGAAGCTTGACGGCAACTGCCTGGTGTATAACATCAAG
ATTCTGGGTTGCAATTTCCCACCGAACGGCCCGGTGATGCAGAAGAAAACACAGGGATGGGAA
CCCTGTTGCGAGATGCGTTATACACGTGATGGAGTATTGTGTGGGCAGACACTTATGGCGCTC
AAATGTGCAGATGGGAACCACCTCACTTGCCACTTACGCACGACGTACCGCTCCAAAAAAGCG
GCAAAGGCGCTGCAGATGCCGCCGTTCCATTTCAGTGACTCACGCCCTGAGATCGTGAAAGTT
AGCGAGAATGGAACTCTTTTCGAACAGCATGAATCCTCAGTGGCCCGCTATTGCCAAACGTGT
CCTTCCAAACTCGGCCATAACTAATAA

NahR

ATGGAACTGCGTGACCTTGATTTAAACCTGCTGGTGGTGTTCAACCAGTTGCTGGTCGACAGA
CGCGTCTCTGTCACTGCGGAGAACCTGGGCCTGACCCAGCCTGCCGTGAGCAATGCGCTGAAA
CGCCTGCGCACCTCGCTACAGGACCCACTCTTCGTGCGCACACATCAGGGAATGGAACCCACA
CCCTATGCCGCGCATCTGGCCGAGCACGTCACTTCGGCCATGCACGCACTGCGCAACGCCCTA
CAGCACCATGAAAGCTTCGATCCGCTGACCAGCGAGCGTACCTTCACCCTGGCCATGACCGAC
ATTGGCGAGATCTACTTCATGCCGCGGCTGATGGATGCGCTGGCTCACCAGGCCCCCAATTGC
GTGATCAGTACGGTGCGCGACAGTTCGATGAGCCTGATGCAGGCCTTGCAGAACGGAACCGTG
GACTTGGCCGTGGGCCTGCTTCCCAATCTGCAAACTGGCTTCTTTCAGCGCCGGCTGCTCCAG
AATCACTACGTGTGCCTATGTCGCAAGGACCATCCAGTCACCCGCGAACCCCTGACTCTGGAG
CGCTTCTGTTCCTACGGCCACGTGCGTGTCATCGCCGCTGGCACCGGCCACGGCGAGGTGGAC
ACGTACATGACACGGGTCGGCATCCGGCGCGACATCCGTCTGGAAGTGCCGCACTTCGCCGCC
GTTGGCCACATCCTCCAGCGCACCGATCTGCTCGCCACTGTGCCGATATGTTTAGCCGACTGC
TGCGTAGAGCCCTTCGGCCTAAGCGCCTTGCCGCACCCAGTCGTCTTGCCTGAAATAGCCATC
AACATGTTCTGGCATGCGAAGTACCACAAGGACCTAGCCAATATTTGGTTGCGGCAACTGATG
TTTGACCTGTTTACGGATTGATAA

RelA+

ATGGTTGCGGTAAGAAGTGCACATATCAATAAGGCTGGTGAATTTGATCCGGAAAAATGGATC
GCAAGTCTGGGTATTACCAGCCAGAAGTCGTGTGAGTGCTTAGCCGAAACCTGGGCGTATTGT
CTGCAACAGACGCAGGGGCATCCGGATGCCAGTCTGTTATTGTGGCGTGGTGTTGAGATGGTG
GAGATCCTCTCGACATTAAGTATGGACATTGACACGCTGCGGGCGGCGCTGCTTTTCCCTCTG
GCGGATGCCAACGTAGTCAGCGAAGATGTGCTGCGTGAGAGCGTCGGTAAGTCGGTCGTTAAC
CTTATTCACGGCGTGCGTGATATGGCGGCGATCCGCCAGCTGAAAGCGACGCACACTGATTCT
GTTTCCTCCGAACAGGTCGATAACGTTCGCCGGATGTTATTGGCGATGGTCGATGATTTTCGC
TGCGTAGTCATCAAACTGGCGGAGCGTATTGCTCATCTGCGCGAAGTAAAAGATGCGCCGGAA
GATGAACGTGTACTGGCGGCAAAAGAGTGTACCAACATCTACGCACCGCTGGCTAACCGTCTC
GGAATCGGACAACTGAAATGGGAACTGGAAGATTACTGCTTCCGTTACCTCCATCCAACCGAA
TACAAACGAATTGCCAAACTGCTGCATGAACGGCGTCTCGACCGCGAACACTACATCGAAGAG
TTCGTTGGTCATCTGCGCGCTGAGATGAAAGCTGAAGGCGTTAAAGCGGAAGTGTATGGTCGT
CCGAAACACATCTACAGCATCTGGCGTAAAATGCAGAAAAAGAACCTCGCCTTTGATGAGCTG
TTTGATGTGCGTGCGGTACGTATTGTCGCCGAGCGTTTACAGGATTGCTATGCCGCACTGGGG
ATAGTGCACACTCACTATCGCCACCTGCCGGATGAGTTTGACGATTACGTCGCTAACCCGAAA
CCAAACGGTTATCAGTCTATTCATACCGTGGTTCTGGGGCCGGGTGGAAAAACCGTTGAGATC
CAAATCCGCACCAAACAGATGCATGAAGATGCAGAGTTGGGTGTTGCTGCGCACTGGAAATAT
AAAGAGGGCGCGGCTGCTGGCGGCGCACGTTCGGGACATGAAGACCGGATTGCCTGGCTGCG
TAAACTGATTGCGTGGCAGGAAGAGATGGCTGATTCCGGCGAAATGCTCGACGAAGTACGTAG
TCAGGTCTTTGACGACCGGGTGTACGTCTTTACGCCGAAAGGTGATGTCGTTGATTTGCCTGC
GGGATCAACGCCGCTGGACTTCGCTTACCACATCCACAGTGATGTCGGACACCGCTGCATCGG
GGCAAAAATTGGCGGGCGCATTGTGCCGTTCACCTACCAGCTG

dCas9

ATGGATAAGAAATACTCAATAGGCTTAGCTATCGGCACAAATAGCGTCGGATGGGCGGTGATC
ACTGATGAATATAAGGTTCCGTCTAAAAAGTTCAAGGTTCTGGGAAATACAGACCGCCACAGT
ATCAAAAAAAATCTTATAGGGGCTCTTTTATTTGACAGTGGAGAGACAGCGGAAGCGACTCGT
CTCAAACGGACAGCTCGTAGAAGGTATACACGTCGGAAGAATCGTATTTGTTATCTACAGGAG
ATTTTTTCAAATGAGATGGCGAAAGTAGATGATAGTTTCTTTCATCGACTTGAAGAGTCTTTT
TTGGTGGAAGAAGACAAGAAGCATGAACGTCATCCTATTTTTGGAAATATAGTAGATGAAGTT
GCTTATCATGAGAAATATCCAACTATCTATCATCTGCGAAAAAAATTGGTAGATTCTACTGAT
AAAGCGGATTTGCGCTTAATCTATTTGGCCTTAGCGCATATGATTAAGTTTCGTGGTCATTTT
TTGATTGAGGGAGATTTAAATCCTGATAATAGTGATGTGGACAAACTATTTATCCAGTTGGTA
CAAACCTACAATCAATTATTTGAAGAAAACCCTATTAACGCAAGTGGAGTAGATGCTAAAGCG
ATTCTTTCTGCACGATTGAGTAAATCAAGACGATTAGAAAATCTCATTGCTCAGCTCCCCGGT
GAGAAGAAAAATGGCTTATTTGGGAATCTCATTGCTTTGTCATTGGGTTTGACCCCTAATTTT
AAATCAAATTTTGATTTGGCAGAAGATGCTAAATTACAGCTTTCAAAAGATACTTACGATGAT
GATTTAGATAATTTATTGGCGCAAATTGGAGATCAATATGCTGATTTGTTTTTGGCAGCTAAG
AATTTATCAGATGCTATTTTACTTTCAGATATCCTAAGAGTAAATACTGAAATAACTAAGGCTC
CCCTATCAGCTTCAATGATTAAACGCTACGATGAACATCATCAAGACTTGACTCTTTTAAAAGC
TTTAGTTCGACAACAACTTCCAGAAAAGTATAAAGAAATCTTTTTTGATCAATCAAAAAACGG
ATATGCAGGTTATATTGATGGGGGAGCTAGCCAAGAAGAATTTTATAAATTTATCAAACCAAT
TTTAGAAAAAATGGATGGTACTGAGGAATTATTGGTGAAACTAAATCGTGAAGATTTGCTGCG
CAAGCAACGGACCTTTGACAACGGCTCTATTCCCCATCAAATTCACTTGGGTGAGCTGCATGC
TATTTTGAGAAGACAAGAAGACTTTTATCCATTTTTAAAAGACAATCGTGAGAAGATTGAAAA
AATCTTGACTTTTCGAATTCCTTATTATGTTGGTCCATTGGCGCGTGGCAATAGTCGTTTTGC
ATGGATGACTCGGAAGTCTGAAGAAACAATTACCCCATGGAATTTTGAAGAAGTTGTCGATAA
AGGTGCTTCAGCTCAATCATTTATTGAACGCATGACAAACTTTGATAAAAATCTTCCAAATGA
AAAAGTACTACCAAAACATAGTTTGCTTTATGAGTATTTTACGGTTTATAACGAATTGACAAA
GGTCAAATATGTTACTGAAGGAATGCGAAAACCAGCATTTCTTTCAGGTGAACAGAAGAAAGC
CATTGTTGATTTACTCTTCAAAACAAATCGAAAAGTAACCGTTAAGCAATTAAAAGAAGATTA
TTTCAAAAAAATAGAATGTTTTGATAGTGTTGAAATTTCAGGAGTTGAAGATAGATTTAATGC
TTCATTAGGTACCTACCATGATTTGCTAAAAATTATTAAAGATAAAGATTTTTTGGATAATGA
AGAAAATGAAGATATCTTAGAGGATATTGTTTTAACATTGACCTTATTTGAAGATAGGGAGAT
GATTGAGGAAAGACTTAAAACATATGCTCACCTCTTTGATGATAAGGTGATGAAACAGCTTAA
ACGTCGCCGTTATACTGGTTGGGGACGTTTGTCTCGAAAATTGATTAATGGTATTAGGGATAA
GCAATCTGGCAAAACAATATTAGATTTTTTGAAATCAGATGGTTTTGCCAATCGCAATTTTAT
GCAGCTGATCCATGATGATAGTTTGACATTTAAAGAAGACATTCAAAAAGCACAAGTGTCTGG
ACAAGGCGATAGTTTACATGAACATATTGCAAATTTAGCTGGTAGCCCTGCTATTAAAAAAGG
TATTTTACAGACTGTAAAAGTTGTTGATGAATTGGTCAAAGTAATGGGGCGGCATAAGCCAGA
AAATATCGTTATTGAAATGGCACGTGAAAATCAGACAACTCAAAAGGGCCAGAAAAATTCGCG
AGAGCGTATGAAACGAATCGAAGAAGGTATCAAAGAATTAGGAAGTCAGATTCTTAAAGAGCA
TCCTGTTGAAAATACTCAATTGCAAAATGAAAAGCTCTATCTCTATTATCTCCAAAATGGAAG
AGACATGTATGTGGACCAAGAATTAGATATTAATCGTTTAAGTGATTATGATGTCGATGCCAT
TGTTCCACAAAGTTTCCTTAAAGACGATTCAATAGACAATAAGGTCTTAACGCGTTCTGATAA
AAATCGTGGTAAATCGGATAACGTTCCAAGTGAAGAAGTAGTCAAAAAGATGAAAAACTATTG
GAGACAACTTCTAAACGCCAAGTTAATCACTCAACGTAAGTTTGATAATTTAACGAAAGCTGA
ACGTGGAGGTTTGAGTGAACTTGATAAAGCTGGTTTTATCAAACGCCAATTGGTTGAAACTCG
CCAAATCACTAAGCATGTGGCACAAATTTTGGATAGTCGCATGAATACTAAATACGATGAAAA
TGATAAACTTATTCGAGAGGTTAAAGTGATTACCTTAAAATCTAAATTAGTTTCTGACTTCCG
AAAAGATTTCCAATTCTATAAAGTACGTGAGATTAACAATTACCATCATGCCCATGATGCGTA
TCTAAATGCCGTCGTTGGAACTGCTTTGATTAAGAAATATCCAAAACTTGAATCGGAGTTTGT
CTATGGTGATTATAAAGTTTATGATGTTCGTAAAATGATTGCTAAGTCTGAGCAAGAAATAGG
CAAAGCAACCGCAAAATATTTCTTTTACTCTAATATCATGAACTTCTTCAAAACAGAAATTACA
CTTGCAAATGGAGAGATTCGCAAACGCCCTCTAATCGAAACTAATGGGGAAACTGGAGAAATT
GTCTGGGATAAAGGGCGAGATTTTGCCACAGTGCGCAAAGTATTGTCCATGCCCCAAGTCAAT
ATTGTCAAGAAAACAGAAGTACAGACAGGCGGATTCTCCAAGGAGTCAATTTTACCAAAAAGA
AATTCGGACAAGCTTATTGCTCGTAAAAAAGACTGGGATCCAAAAAAATATGGTGGTTTTGAT
AGTCCAACGGTAGCTTATTCAGTCCTAGTGGTTGCTAAGGTGGAAAAAGGGAAATCGAAGAAG
TTAAAATCCGTTAAAGAGTTACTAGGGATCACAATTATGGAAAGAAGTTCCTTTGAAAAAAAT
CCGATTGACTTTTTAGAAGCTAAAGGATATAAGGAAGTTAAAAAAGACTTAATCATTAAACTA
CCTAAATATAGTCTTTTTGAGTTAGAAAACGGTCGTAAACGGATGCTGGCTAGTGCCGGAGAA
TTACAAAAAGGAAATGAGCTGGCTCTGCCAAGCAAATATGTGAATTTTTTATATTTAGCTAGT
CATTATGAAAAGTTGAAGGGTAGTCCAGAAGATAACGAACAAAAACAATTGTTTGTGGAGCAG
CATAAGCATTATTTAGATGAGATTATTGAGCAAATCAGTGAATTTTCTAAGCGTGTTATTTTA
GCAGATGCCAATTTAGATAAAGTTCTTAGTGCATATAACAAACATAGAGACAAACCAATACGT
GAACAAGCAGAAAATATTATTCATTTATTTACGTTGACGAATCTTGGAGCTCCCGCTGCTTTT
AAATATTTTGATACAACAATTGATCGTAAACGATATACGTCTACAAAAGAAGTTTTAGATGCC
ACTCTTATCCATCAATCCATCACTGGTCTTTATGAAACACGCATTGATTTGAGTCAGCTAGGA
GGTGACTAA

p15A

TTGAGATCGTTTTGGTCTGCGCGTAATCTCTTGCTCTGAAAACGAAAAAACCGCCTTGCAGGG
CGGTTTTTCGAAGGTTCTCTGAGCTACCAACTCTTTGAACCGAGGTAACTGGCTTGGAGGAGC
GCAGTCACCAAAACTTGTCCTTTCAGTTTAGCCTTAACCGGCGCATGACTTCAAGACTAACTCC
TCTAAATCAATTACCAGTGGCTGCTGCCAGTGGTGCTTTTGCATGTCTTTCCGGGTTGGACTC
AAGACGATAGTTACCGGATAAGGCGCAGCGGTCGGACTGAACGGGGGGTTCGTGCATACAGTC
CAGCTTGGAGCGAACTGCCTACCCGGAACTGAGTGTCAGGCGTGGAATGAGACAAACGCGGCC
ATAACAGCGGAATGACACCGGTAAACCGAAAGGCAGGAACAGGAGAGCGCACGAGGGAGCCG
CCAGGGGGAAACGCCTGGTATCTTTATAGTCCTGTCGGGTTTCGCCACCACTGATTTGAGCGT
CAGATTTCGTGATGCTTGTCAGGGGGGCGGAGCCTATGGAAA

Amp

CACCCAGAAACGCTGGTGAAAGTAAAAGATGCTGAAGATCAGTTGGGTGCACGAGTGGGTTAC
ATCGAACTGGATCTCAACAGCGGTAAGATCCTTGAGAGTTTTCGCCCCGAAGAACGTTTTCCA
ATGATGAGCACTTTTAAAGTTCTGCTATGTGGCGCGGTATTATCCCGTATTGACGCCGGGCAA
GAGCAACTCGGTCGCCGCATACACTATTCTCAGAATGACTTGGTTGAGTACTCACCAGTCACA
GAAAAGCATCTTACGGATGGCATGACAGTAAGAGAATTATGCAGTGCTGCCATAACCATGAGT
GATAACACTGCGGCCAACTTACTTCTGACAACGATCGGAGGACCGAAGGAGCTAACCGCTTTT
TTGCACAACATGGGGGATCATGTAACTCGCCTTGATCGTTGGGAACCGGAGCTGAATGAAGCC
ATACCAAACGACGAGCGTGACACCACGATGCCTGTAGCAATGGCAACAACGTTGCGCAAACTA
TTAACTGGCGAACTACTTACTCTAGCTTCCCGGCAACAATTAATAGACTGGATGGAGGCGGAT
AAAGTTGCAGGACCACTTCTGCGCTCGGCCCTTCCGGCTGGCTGGTTTATTGCTGATAAATCT
GGAGCCGGTGAGCGTGGGTCTCGCGGTATCATTGCAGCACTGGGGCCAGATGGTAAGCCCTCC
CGTATCGTAGTTATCTACACGACGGGGAGTCAGGCAACTATGGATGAACGAAATAGACAGATC
GCTGAGATAGGTGCCTCACTGATTAAGCATTGGTAA

Table 2: Essential DNA sequences used in this study.

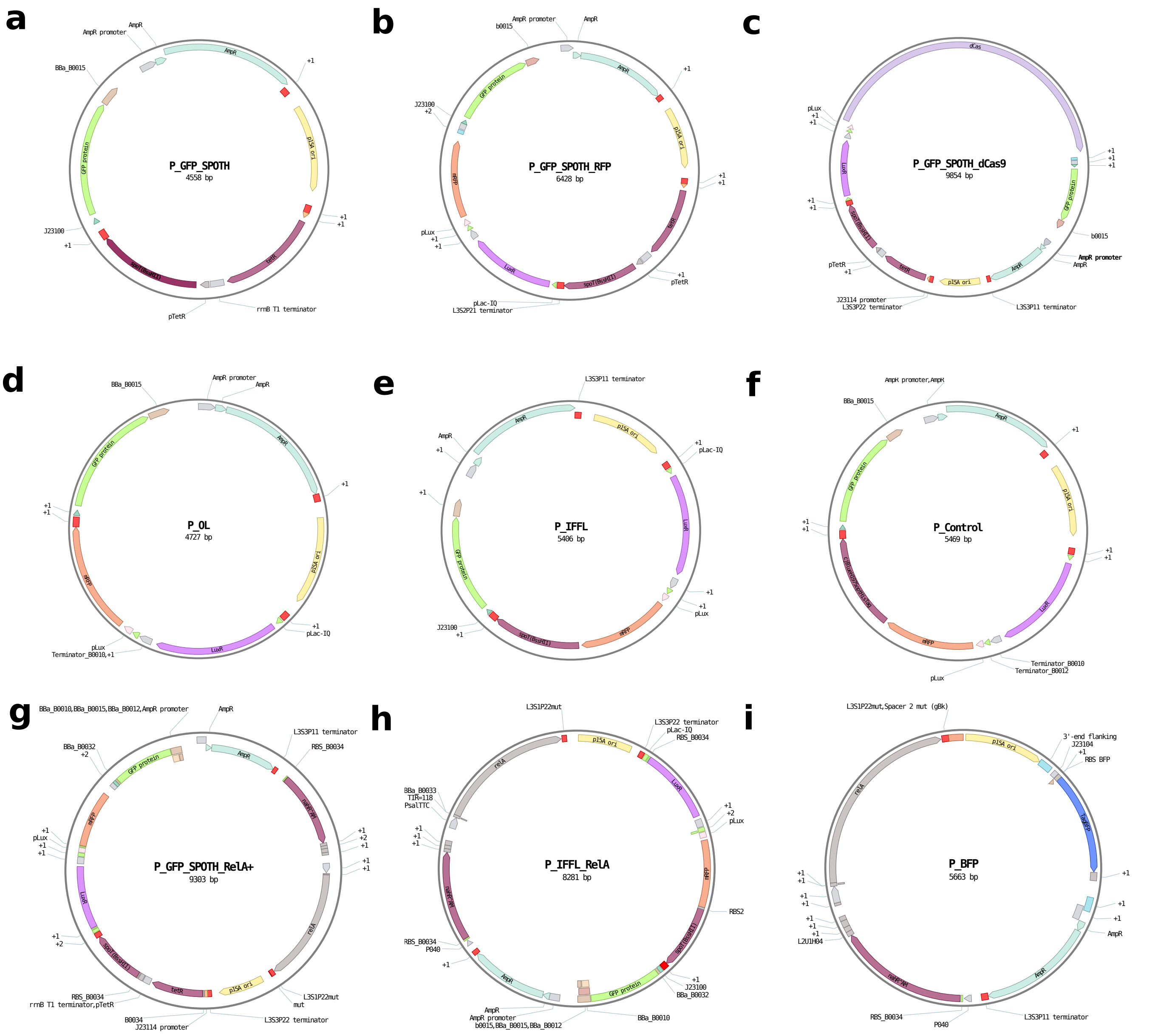

Supplementary Figure. 11: **Plasmid maps** The plasmid maps were prepared by the Benchling Life Sciences R&D platform. **(a)** P__GFP__SpoTH **(b)** P__FP__SpoTH__RFP **(c)** P__GFP__SpoTH__dCas9 **(d)** P__OL **(e)** P__IFFL__x **(f)** P__Control **(g)** P__GFP__SpoTH__RelA+ **(h)** P__IFFL__RelA__x **(i)** P__BFP

Supplementary note 1

The SpoTH gene sequence was constructed based on the BssHII digestion and re-ligation of the spoT gene (pGN19 in [21]), which was shown to only have ppGppasse activity. The digestion and re-ligation of spoT using BssHII introduces a frameshift following the 206 codon and consequently a premature stop codon after the 217 codon. Therefore, the SpoTH sequence only contains the first 217 condons of the product of re-ligating and digesting spoT using BssHII. Finally we modified the initial codon of

the endogenous spoT gene from TTG to ATG. The full SpoTH sequence is shown in Supplementary Table 2.

Supplementary note 2

Summary of simplified SpoTH actuator model: Here, we provide a simplified mathematical model that describes how expressing SpoTH actuates free ribosome concentration. The full model derivation and details can be found in Section: *Derivation of the SpoTH actuator mathematical model.* A key component of the model is the total ribosome concentration equation given by

$$R_T = R + c_s + c_e, \tag{21}$$

where R_T is the concentration of the total ribosomes in the cell, R is the concentration of free ribosomes, and c_s and c_e are the concentrations of the mRNA-ribosome complex corresponding to SpoTH mRNA and the mRNA corresponding to the cell's endogenous genes, respectively. The concentration of SpoTH is proportional to c_s (3) and thus from hereon, we use varying SpoTH expression and varying c_s interchangeably. Let c_e be a general function of free ribosome concentration, that is, $c_e := c_e(R)$. We assume that more endogenous mRNA is translated when R increases, that is, $\frac{dc_e}{dR} > 0$. We define

$$z(R) = R + c_e(R), \tag{22}$$

which satisfies $z(0) = 0$ (in the absence of free ribosomes no endegnous mRNA is translated) and is monotonically increasing with R. We rewrite (21) using (22), using a model of how R_T depends on c_s through SpoTH catalyzing ppGpp hydrolysis (see Section: *Actuating the ribosomal budget in the cell*), and using overbars to denote concentrations normalized by the total ribosome concentration when there is no ppGpp in the cell, as:

$$\bar{R}_T = \bar{z}_0 + (1 - \bar{z}_0) f(\bar{c}_s/\epsilon, \bar{z}_0) = \bar{z} + \bar{c}_s, \tag{23}$$

where $\bar{z}_0 \in [0, 1]$ is a proxy of the nominal free ribosome concentration corresponding to no SpoTH expression ($\bar{z}_0 := \bar{z}|_{\bar{c}_s=0}$), $f \in [0, 1)$ is given by $f = \frac{(\bar{c}_s/\epsilon)(\bar{c}_s/\epsilon+2)}{(\bar{c}_s/\epsilon)(\bar{c}_s/\epsilon+2)+1/\bar{z}_0}$ and captures how SpoTH increases the total ribosome concentration, and ϵ is a dimensionless parameter that measures how effectively SpoTH catalyzes the hydrolysis of ppGpp and how effectively SpoTH-mRNA is translated into protein. An additional key quantity is

$$\delta(\bar{z}_0, \epsilon) := \left.\frac{d\bar{z}}{d\bar{c}_s}\right|_{\bar{c}_s=0} = 2\frac{\bar{z}_0(1 - \bar{z}_0)}{\epsilon} - 1, \tag{24}$$

which is the slope of $\bar{z}$ at $c_s = 0$. The qualitative behavior of (23) is shown in Supplementary Fig. 12 and it has three qualitatively different responses. When $\delta > 0$, we obtain a desired actuator profile where $\bar{z}$ increases initially as $\bar{c}_s$ increases, as $\bar{c}_s$ continues to increase the f term saturates to unity and the right hand side $\bar{c}_s$ term of (23) dominates and thus the actuator profile peaks and then decreases. As $\delta \to \infty$, the peak actuation and actuator operational range both approach the quantity $(1 - \bar{z}_0)$. When $\delta < 0$, $\bar{z}$ decreases initially as $\bar{c}_s$ increases and then it can either continue to decrease or it can eventually increase past $\bar{z}_0$, peak, and then decrease again.

Remark 1. From (24), for a fixed ϵ such that $\epsilon < 0.5$, there exists $0 < \bar{z}_{0,*}(\epsilon) < \bar{z}_0^*(\epsilon) < 1$ such that for all $\bar{z}_0 \in (\bar{z}_{0,*}, \bar{z}_0^*)$ we have that $\delta > 0$ and for all $\bar{z}_0$ outside this set, $\delta < 0$. In (13) we show that $\bar{z}_0$ monotonically decreases with basal ppGpp (θ_G) and thus for a fixed ϵ, there is an open interval of basal ppGpp values that render the desired actuation profile. This implies that too high or too low basal ppGpp can be detrimental in achieving the desired actuator profile.

In Supplementary Fig. 13, we show the normalized actuation ($\bar{z}/\bar{z}_0$) profile for several $\bar{z}_0$ values. We observe that for lower $\bar{z}_0$ we have more normalized peak actuation. In the inset we show that the normalized peak actuation increases with $\bar{z}_0$ up until $\bar{z}_0 \approx 0.1$. After this critical value, peak actuation decreases as $\bar{z}_0$ decreases.

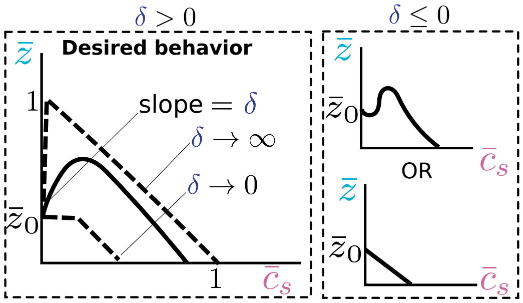

Supplementary Figure. 12: **Qualitative behavior of actuator.** For $\delta > 0$ (24), the actuator profile predicted by (23) has the desired behavior where $\bar{z}$ (proxy of free ribosomes) increases as SpoTH is expressed (increasing $\bar{c}_s$), then it peaks and begins to drop. The asymptotic behavior as $\delta \to \infty$ and $\delta \to 0$ are depicted by dashed lines. When $\delta < 0$, $\bar{z}$ initially decreases as SpoTH is expressed. It can then either continue to decrease or at some point increase, peak, and then decrease again.

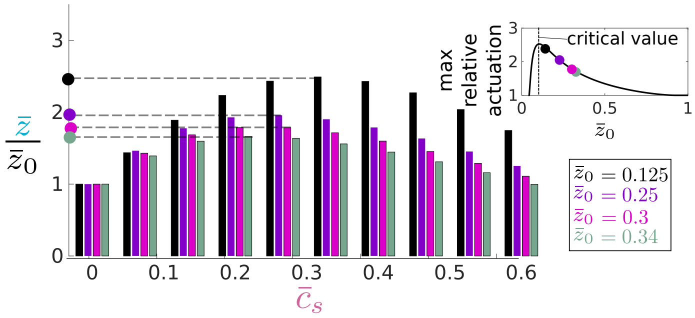

Supplementary Figure. 13: **Tradeoff between nominal level and normalized peak actuation.** The actuation profile predicted by (23) for several $\bar{z}_0$. We observe that for lower $\bar{z}_0$, there is higher normalized peak actuation. In the inset we show that the normalized actuation increases as $\bar{z}_0$ increases up until the critical value of $\bar{z}_0 \approx 0.1$. After this critical value normalized peak actuation decreases as $\bar{z}_0$ increases. For this simulation we have $\epsilon = 0.13$.

Simplified model of expressing a heterologous protein: When accounting for the expression of a heterologous protein y, the total ribosome concentration equation (21) is modified to

$$R_T = R + c_s + c_y + c_e, \tag{25}$$

where c_y is the concentration of the mRNA-ribosome complex corresponding to the mRNA of y. The protein concentration of y is proportional to c_y. The dimensionless total ribosome equation (23) now reads

$$\bar{z}_0 + (1 - \bar{z}_0) f(\bar{c}_s/\epsilon, \bar{z}_0) = \bar{z} + \bar{c}_s + \bar{c}_y. \tag{26}$$

In Supplementary Section: *Derivation of the SpoTH actuator mathematical model*, we show that at steady state, the quantities $\bar{c}_y$ and $\bar{c}_s$ are given by

$$\bar{c}_y = m_y \bar{R}/K_y, \quad \bar{c}_s = m_s \bar{R}/K_s, \tag{27}$$

where m_y is the y mRNA concentration, K_y is the dissociation constant of free ribosome with y mRNA, m_s is the SpoTH mRNA concentration, and K_s is the dissociation constant of free ribosome with SpoTH mRNA. Each of K_y and K_s can be tuned by changing the ribosome binding site (RBS) of the corresponding mRNA. From (27), we have that

$$\bar{c}_s = \frac{m_s}{m_y} \gamma \bar{c}_y, \quad \gamma = K_y/K_s, \tag{28}$$

where γ is the ratio between the SpoTH RBS strength and the y RBS strength.

Feedforward controller: We model SpoTH and y as being transcribed from the same promoter, which implies $m_s = m_y$. We refer to the configuration where y and SpoTH are transcriptionally coupled this way as the closed loop system and it obeys (26) with $\bar{c}_s = \gamma\bar{c}_y$ as shown by (28) when $m_s = m_y$. We denote expressing y in the absence of SpoTH as the open loop system and it obeys (26) with $\bar{c}_s = 0$ for all values of $\bar{c}_y$. The qualitative behavior of the closed loop system compared to the open loop system is shown in Supplementary Fig. 10. We define the ideal relationship between $\bar{z}$ and $\bar{c}_y$ as $\bar{z} = \bar{z}_0$ for all $\bar{c}_y$, as shown in Supplementary Fig. 14. The initial slope ($\frac{d\bar{z}}{d\bar{c}_y}|_{\bar{c}_y=0}$) of the closed loop system is given by $\gamma\delta - 1$, where δ is given by (24). Thus, if $\delta > 0$, which, from Fig 12, implies that we are in the parameter regime such that the actuator has a desired profile, then the SpoTH RBS strength (γ) can be chosen such that $\gamma(\bar{z}_0, \epsilon) = 1/\delta(\bar{z}_0, \epsilon)$ to render an initial flat response of $\bar{z}$ as $\bar{c}_y$ increases. In Supplementary Fig. 14, we show the closed loop system response (blue lines) for $\gamma < 1/\delta$, $\gamma = 1/\delta$, and $\gamma > 1/\delta$ and the open loop system response (orange). As expected, for $\gamma = 1/\delta$, the response of $\bar{z}$ is initially flat as $\bar{c}_y$ is expressed. The closed loop system achieves higher values of $\bar{z}$ than the open loop system. Furthermore, we observe that the closed loop system achieves higher values of c_y than the open loop system.

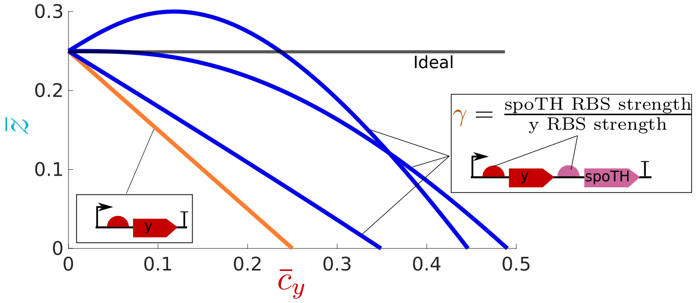

Supplementary Figure. 14: **Feedforward controller to compensate for the burden on ribosomes caused by heterologous protein overexpression.** Simulation of (26) with $\bar{c}_s = \gamma\bar{c}_y$. This corresponds to placing y and SpoTH under same promoter (closed loop) depicted in blue. The SpoTH RBS (γ) can be tuned to approximate the ideal scenario where $\bar{z} = \bar{z}_0$ for all $\bar{c}_y$. We also show the open loop system (y without SpoTH) depicted in orange as given by (26) with $c_s = 0$. For this simulation we have $\bar{z}_0 = 0.25$ and $\epsilon = 0.13$. For the closed loop, we have that $\gamma = 0.16, 0.53, 0.9$.

Supplementary note 3

The model from Supplementary note 2 relates SpoTH expression to free ribosome concentration (or equivalently z), here we propose a model to relate SpoTH expression to the cell growth rate (μ). A precise model of growth rate as SpoTH is expressed would require a whole-cell model [24]. However, in this work we are interested in the qualitative behavior of growth rate. Thus, we don't consider an explicit model and rather assume that the growth rate is given by

$$\mu := h(G, R) \tag{29}$$

with the properties that $\frac{\partial h}{\partial G} \leq 0$, $\frac{\partial h}{\partial R} \geq 0$, and that $h(G, 0) = 0$. The relationship (29) is consistent with the interaction diagram from Fig. 1-a in the main text where ppGpp directly downderegulates growth genes and thus growth rate ($\frac{\partial h}{\partial G} \leq 0$) and free ribosome translates mRNA's responsible for cell growth and thus they upregulate growth rate ($\frac{\partial h}{\partial R} \geq 0$). Furthermore, $h(G, 0) = 0$ implies that cells cannot grow when there are no free ribosomes present, which is consistent with physical intuition.

Growth rate versus the SpoTH gene activation. The change in growth rate as SpoTH is

expressed, is given by

$$\frac{dh}{dS} = \frac{\partial h}{\partial G}\frac{dG}{dS} + \frac{\partial h}{\partial R}\frac{dR}{dS}, \tag{30}$$

per (6) we have that

$$\frac{dG}{dS} = -\frac{G_0/K_{gs}}{(1+S/K_{gs})^2} \implies \frac{dG}{dS} \leq 0 \tag{31}$$

and $\frac{dR}{dS}$ is how free ribosome concentration changes as SpoTH is expressed. From (29) and (31) and our assumptions on h, we have that the quantities $\frac{\partial h}{\partial G}\frac{dG}{dS}$ and $\frac{\partial h}{\partial R}$ are positive, implying that:

- The mapping between growth rate and SpoTH expression is qualitatively similar to that of Supplementary Fig. 12 except that the growth rate peak occurs at a higher SpoTH expression level than the peak in free ribosomes. This is consistent with our experimental data where GFP production rate peaked (Supplementary Fig. 1) for lower values of SpoTH (aTc) compared to growth rate (Main text Fig. 2). The data and (30) is also consistent with the fact that growth rate can increase with SpoTH expression while free ribosomes decrease with SpoTH expression. The assumption that $h(G, 0) = 0$ implies that the growth rate indeed reaches a maximum when SpoTH is expressed and then approaches zero as the SpoTH mRNA sequesters all the available ribosomes.

- ppGpp levels can be used to tune the mapping between growth rate and SpoTH expression in a similar matter as for free ribosome concentration is tuned (see Remark 1).

Growth rate in feedforward controller. In a feedforward configuration we have that the protein level of the GOI (y) is proportional to that of SpoTH, that is $y = \theta_y S$ (Supplementary note 2), where θ_y is a positive constant. Thus, the change in growth rate as the GOI is expressed in the feedforward configuration is given by

$$\frac{dh}{dy} = \frac{\partial h}{\partial G}\frac{dG}{dS}\frac{dS}{dy} + \frac{\partial h}{\partial R}\frac{dR}{dy} = \theta_y\frac{\partial h}{\partial G}\frac{dG}{dS} + \frac{\partial h}{\partial R}\frac{dR}{dy}. \tag{32}$$

From (31), our assumptions on h, and the fact that $\theta_y > 0$, we have that the mapping between growth rate and GOI expression is qualitatively similar to that of Supplementary Fig. 14 and the SpoTH RBS can be used to make growth rate initially flat as the GOI is expressed. However, the SpoTH RBS that makes growth rate flat ($\frac{dh}{dy}|_{y=0} = 0$) is one where $\frac{dR}{dy} < 0$, that is, free ribosome decrease with GOI expression. This is consistent with our experimental data where GFP production rate decreases with GOI expression (Supplementary Fig. 3) and growth rate is nearly flat (Main text Fig. 3).

Supplementary note 4

Through a simple mathematical model we show that the protein production rate of a constitutive protein is a proxy for free ribosome concentration. This is consistent with [25] where the constitutive expression of a GFP monitor was used as a proxy for free ribosome levels.

We model mRNA (m) binding to free ribosomes (R) to produce the translation initiation complex c, which is then translated to produce the protein P with elongation rate constant κ. The mRNA decays with rate constants δ and the protein dilutes with rate constant γ. The corresponding chemical reactions are:

$$\emptyset \xrightarrow{\alpha_{\mathrm{m}}} \mathrm{m}, \quad \mathrm{m} \xrightarrow{\delta} \emptyset, \quad \mathrm{m} + \mathrm{R} \underset{\mathrm{d}}{\overset{\mathrm{a}}{\rightleftharpoons}} \mathrm{c} \xrightarrow{\kappa} \mathrm{m} + \mathrm{R} + \mathrm{P}, \quad \mathrm{P} \xrightarrow{\gamma} \emptyset, \tag{33}$$

where α_m is the production rate constant of the mRNA, a and d are the association and dissociation rate constant, respectively, between ribosomes and mRNA. The concentration of each species satisfies:

$$\begin{aligned} \frac{dm}{dt} =& \alpha_m - aRm + (d+\kappa)c - \delta m, \\ \frac{dc}{dt} =& aRm - (d+\kappa)c \\ \frac{dP}{dt} =& \kappa c - \gamma P. \end{aligned} \tag{34}$$

The ribosome-mRNA dynamics can be assumed to be fast relative to γ [26] and thus the quasi-steady state [20] of (34) is given by

$$m = \frac{\alpha_m}{\delta}, \quad c = \frac{m}{K}R, \tag{35}$$

where $K = \frac{d+\kappa}{a}$. Thus, the reduced protein concentration dynamics are given by

$$\frac{dP}{dt} = \underbrace{\frac{\kappa\alpha_m}{K\delta}R}_{\alpha_P} - \gamma P.$$

where α_P is the protein production rate. If the protein is constitutively expressed, then α_m is constant and α_P is given by a constant ($\frac{\kappa\alpha_m}{K\delta}$) multiplied by R, implying that the protein production rate is a proxy for free ribosome concentration.

Supplementary note 5

Our modeling framework suggests that we can tune the SpoTH RBS strength in the closed loop genetic circuit (express heterologous protein and SpoTH on the same mRNA) to minimize the sensitivity of free ribosomes on heterologous protein expression (Supplementary Fig. 14). Therefore, we created a SpoTH RBS library: RBS 1, RBS 2, RBS3, and RBS 4, to test on the closed loop circuit. In this section we characterize the relative strength of the library in the configuration where SpoTH is expressed on the same mRNA as RFP (placed upstream of SpoTH). We show that the strength of the RBS increases in the following order: RBS 1, RBS 2, RBS 3, and RBS 4.

The RBS strength is dependent on the upstream and downstream sequences of the RBS [27, 28], therefore we characterize the SpoTH RBS library with RFP upstream of SpoTH so that the results are applicable to the closed loop controller (Fig. 3-d in the main text). However, we decrease the RBS strength of RFP by several fold (MBP 0.006 in [5]) such that the amount of ribosomes it sequesters are negligible (relative to SpoTH actuation) and thus the change in ribosome concentration when expressing the mRNA with both the weak RFP RBS and SpoTH, is identical to SpoTH in isolation. The construct used to characterize the SpoTH RBS library is shown in Fig.15-a.

Increasing the RBS strength implies that for a fixed amount of SpoTH mRNA, more ribosomes are recruited to translate the mRNA and thus more SpoTH protein is produced. Therefore, less SpoTH mRNA is needed to actuate as the SpoTH RBS strength increases. This implies that when expressing SpoTH using the construct shown in Fig.15-a, less AHL is needed to see an actuation of GFP production rate and growth rate as the SpoTH RBS increases. The GFP production rate and growth rate data are shown in Fig.15-b and Fig.15-c, respectively, when expressing SpoTH using the

genetic circuit in Fig.15-a with lactose as the carbon source. We observe that for the list: RBS 1, RBS 2, RBS 3, and RBS 4, that the amount of AHL needed to actuate the GFP production rate and growth rate decreases. Thus, based on our physical intuition, it implies that the RBS strength should have an increasing order of: RBS 1, RBS 2, BS 3, and RBS 4. The same trend is observed in Fig.15-d and Fig.15-e when using glycerol as the carbon source.

Our physical intuition that increasing the SpoTH RBS strength implies that less mRNA is needed to see an actuation on free ribosomes, can be made mathematically precise using the actuator model (12), which relates free ribosome concentration to SpoTH expression. From the fact that

$$\bar{c}_s = m_s \bar{R}/K_s,$$

where m_s is the SpoTH mRNA and K_S is inversely proportional to the SpoTH RBS strength, to specify $\bar{c}_s$ we need to know the value $\bar{R}$. Therefore, we need to specify specify $\bar{c}_e(R)$. We assume that $\bar{c}_e(R)$ has a form similar to that of $\bar{c}_s$, then for q different endogenous genes expressing mRNA, $\bar{c}_e(R) = \sum_{i=1}^{q} \frac{m_{e,i}}{K_{e,i}} \bar{R}$, where for gene i, $m_{e,i}$ is the endogenous mRNA concentration and $K_{e,i}$ is the effective dissociation constant of endogenous mRNA with ribosomes. In this cases, $\bar{c}_e(R)$ satisfies all of the assumptions stated in Section: *Derivation of the SpoTH actuator mathematical model.* and furthermore, $\bar{R} = \frac{\bar{z}(R)}{1+\sum_{i=1}^{q} \frac{m_{e,i}}{K_{e,i}}}$. Let $m_s^* = \frac{m_s}{1+\sum_{i=1}^{q} \frac{m_{e,i}}{K_{e,i}}}$ and thus (26) now reads:

$$\bar{z}_0 + (1-\bar{z}_0) f(\bar{z} m_s^*/K_s/\epsilon, \bar{z}_0) = \bar{z} + \bar{z} m_s^*/K_s. \tag{36}$$

The results from simulating (36) are shown in Fig.16. We observe that increasing the RBS strength (decrease K_s) the amount of SpoTH mRNA (m_s^*) needed to actuate $\bar{z}$ decreases.

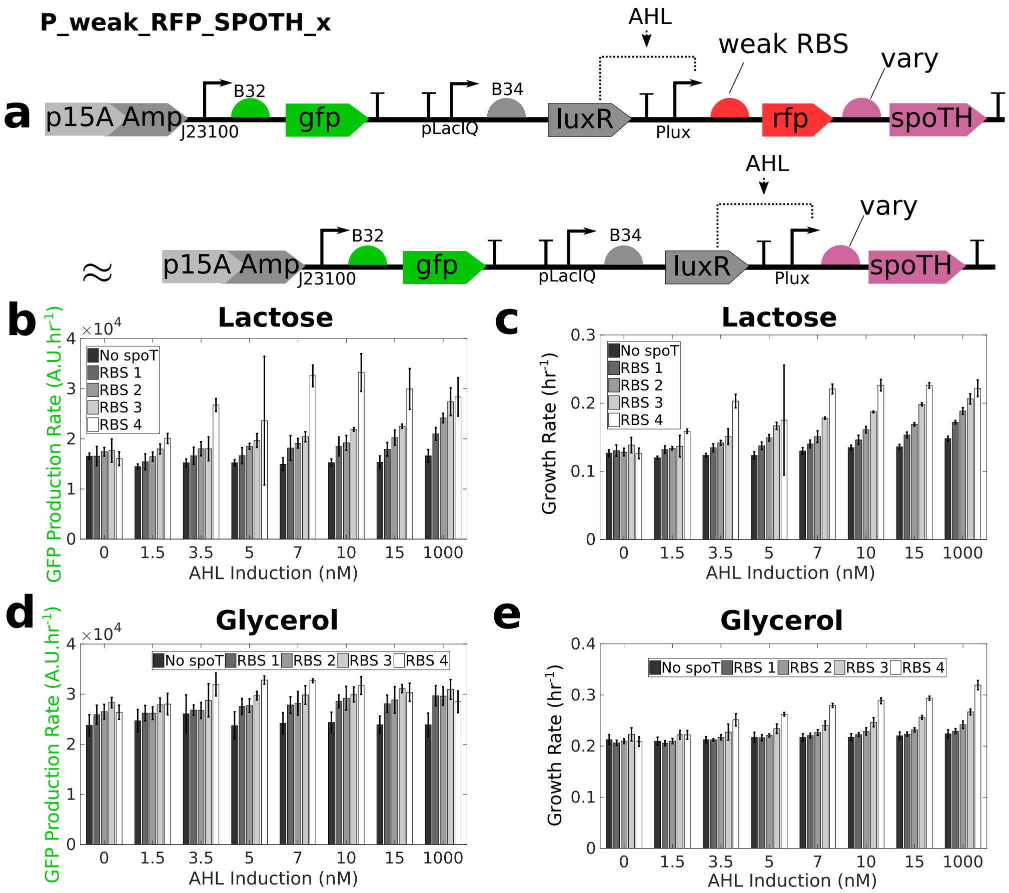

Supplementary Figure. 15: **Characterizing the SpoTH RBS library strengths (a)** The P_weak_RFP_SPOTH_x genetic construct used to characterize the SpoTH RBS library. This construct is identical to Fig. 3-d (P_IFFL_x) in the main text, but with a very weak RFP RBS strength. The genetic construct P_weak_RFP (identical to P_OL with weak RBS for RFP) corresponds to "No SpoTH" in the legends. Plasmid description, plasmid map, and essential DNA sequences are provided in Supplementary section *Plasmid maps and DNA sequences*. **(b)** For lactose as the carbon source, the GFP production rate as SpoTH is expressed (increase AHL) for the RBS library. **(c)** For lactose as the carbon source, the growth rate as SpoTH is expressed (increase AHL) for the RBS library. **(d)** For glycerol as the carbon source, the GFP production rate as SpoTH is expressed (increase AHL) for the RBS library. **(e)** For glycerol as the carbon source, the growth rate as SpoTH is expressed (increase AHL) for the RBS library. For all data, error bars represent standard deviation from at least four replicates (two biological replicates each with two technical replicates). Data are shown as the mean ± one standard deviation (N=4, two biological replicates each with two technical replicates). All experiments were performed in the CF945 strain. The complete experimental protocol is provided in the Materials and Methods section.

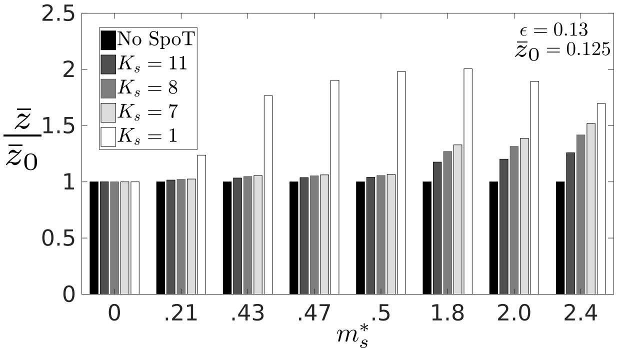

Supplementary Figure. 16: **SpoTH expression with several RBS strengths** The normalized measure of free ribosome concentration $\bar{z}/\bar{z}_0$ predicted by (36) as the normalized SpoTH mRNA m_s^* and SpoTH RBS strength ($1/K_s$) are varied. The simulation parameters are $\epsilon = 0.13$ and $\bar{z}_0 = 0.125$. The "No SpoT" bars correspond to $\bar{z}/\bar{z}_0 = 1$ for all m_s^* values.

Supplementary note 6

For Fig. 5-e in the main text, when the RFP is activated, the growth rate for the OL system in CF946 drops by 40% and that of the associated CL system with SpoTH RBS 2 decreases by 15%. The growth rate for CL RBS 3 corresponding to the set up of Fig. 5-e, monotonically increase by 40% as RFP is activated (Supplementary Fig. 6) thus implying the existence of a CL RBS with strength between RBS 2 and RBS 3 such that the growth rate of the CL systems remains constant as RFP is activated (Supplementary Fig. 14 and Supplementary Note 3).

Supplementary note 7

dCas9 expression is known to be toxic in many bacteria [29, 30]. To this end, we use the SpoTH actuator to reduce growth defects due to overexpressing the dCas9 protein. We express SpoTH using the inducible pTet promoter and dCas9 using the inducible Plux promoter (Supplementary Fig. 17-a). To estimate the relative production rates of dCas9 between induction values and to assess how much of the burden of expressing dCas9 comes from toxicity rather than ribosome sequestration, we replace dCas9 in Supplementary Fig. 17-a with RFP (Supplementary Fig. 17-b). The induction of dCas9 with no SpoTH expression results in a $\sim 40\%$ drop in growth rate (Supplementary Fig. 17-c). For every dCas9 induction level, there is a SpoTH induction that results in a growth rate that is near the nominal value when no dCas9 nor SpoTH are expressed (colored dots in Supplementary Fig. 17-c). For AHL = 0.25 nM, growth rate drops by $\sim 8\%$ when expressing dCas9 and without SpoTH, suggesting that toxicity is already present. However, by expressing SpoTH, even for AHL = 2.0 nM, growth rate stays nearly constant (Supplementary Fig. 17-d). At AHL = 2.0 nM, nearly four times more RFP is produced than at AHL = 0.25 nM (Supplementary Fig. 17-d). The assumption that RFP production rate is proportional to that of the dCas9, implies that four times more dCas9 is produced at AHL = 2.0 nM than at AHL = 0.25 nM. Thus, we conclude that with the appropriate SpoTH expression, we can produce four times the amount of dCas9 that would otherwise be toxic to the cell, while keeping growth rate constant. Additionally, GFP production rate drops by $\sim 40\%$ when dCas9 is expressed and SpoTH, in principle, can be used to keep it constant (Supplementary Fig. 17-e,f).

Expressing RFP with the same AHL values as those tested in Supplementary Fig. 17-c, leads to minimal growth defects but also small drops in GFP production rate when compared to expressing

dCas9 (Supplementary Fig. 17-d,f). Assuming that changes in GFP production rate are a proxy for changes in free ribosome concentration (Supplementary note 4), the incomparable drop in GFP production rate when expressing RFP rather than dCas9 makes it difficult to conclude how much of the burden of expressing dCas9 comes from toxicity rather than ribosome sequestration. To this end, we expressed RFP to a level that would yield a comparable drop in GFP production (more than 40%) as when dCas9 is expressed to the levels in Supplementary Fig. 17-f, and observed that growth rate only dropped by $\sim 15\%$ (Supplementary Fig. 18). This indicates that a large portion of the observed growth defects when expressing dCas9 are likely due to direct toxicity as opposed to being due to ribosome sequestration, consistent with published literature [31, 32, 33].

References

[1] M. C. Chan, S. Karasawa, H. Mizuno, I. Bosanac, D. Ho, G. G. Privé, A. Miyawaki, and M. Ikura, "Structural characterization of a blue chromoprotein and its yellow mutant from the sea anemone Cnidopus japonicus," *J. Biol. Chem.*, vol. 281, no. 49, pp. 37813–37819, 2006.

[2] E. Sarubbi, K. E. Rudd, and M. Cashel, "Basal ppGpp level adjustment shown by new spoT mutants affect steady state growth rates and rrnA ribosomal promoter regulation in Escherichia coli," *MGG Mol. Gen. Genet.*, vol. 213, no. 2-3, pp. 214–222, 1988.

[3] M. Zhu and X. Dai, "Growth suppression by altered (p)ppGpp levels results from non-optimal resource allocation in Escherichia coli," *Nucleic Acids Res.*, vol. 47, no. 9, pp. 4684–4693, 2019.

[4] D. Del Vecchio, R. M. Murray, and D. Vecchio, *Biomolecular Feedback Systems.* 2010.

[5] A. Gyorgy, J. I. Jiménez, J. Yazbek, H. H. Huang, H. Chung, R. Weiss, and D. Del Vecchio, "Isocost Lines Describe the Cellular Economy of Genetic Circuits," *Biophys. J.*, vol. 109, no. 3, pp. 639–646, 2015.

[6] Y. Qian, H. H. Huang, J. I. Jiménez, and D. Del Vecchio, "Resource Competition Shapes the Response of Genetic Circuits," *ACS Synth. Biol.*, vol. 6, no. 7, pp. 1263–1272, 2017.

[7] H. H. Huang, Y. Qian, and D. Del Vecchio, "A quasi-integral controller for adaptation of genetic modules to variable ribosome demand," *Nat. Commun.*, vol. 9, no. 1, pp. 1–12, 2018.

[8] B. J. Paul, W. Ross, T. Gaal, and R. L. Gourse, "rRNA transcription in Escherichia coli," *Annu. Rev. Genet.*, vol. 38, pp. 749–770, 2004.

[9] K. Potrykus and M. Cashel, "Growth at best and worst of times," *Nat. Microbiol.*, vol. 3, no. 8, pp. 862–863, 2018.

[10] A. G. Marr, "Growth Rate of Escherichia coli," *Microbiol. Mol. Biol. Rev.*, vol. 55, no. 2, pp. 316–333, 1991.

[11] H. D. Murray, J. A. Appleman, and R. L. Gourse, "Regulation of the Escherichia coli rrnB P2 promoter," *J. Bacteriol.*, vol. 185, no. 1, pp. 28–34, 2003.

[12] K. Potrykus, H. Murphy, N. Philippe, and M. Cashel, "ppGpp is the major source of growth rate control in E. coli," *Environ. Microbiol.*, vol. 13, no. 3, pp. 563–575, 2011.

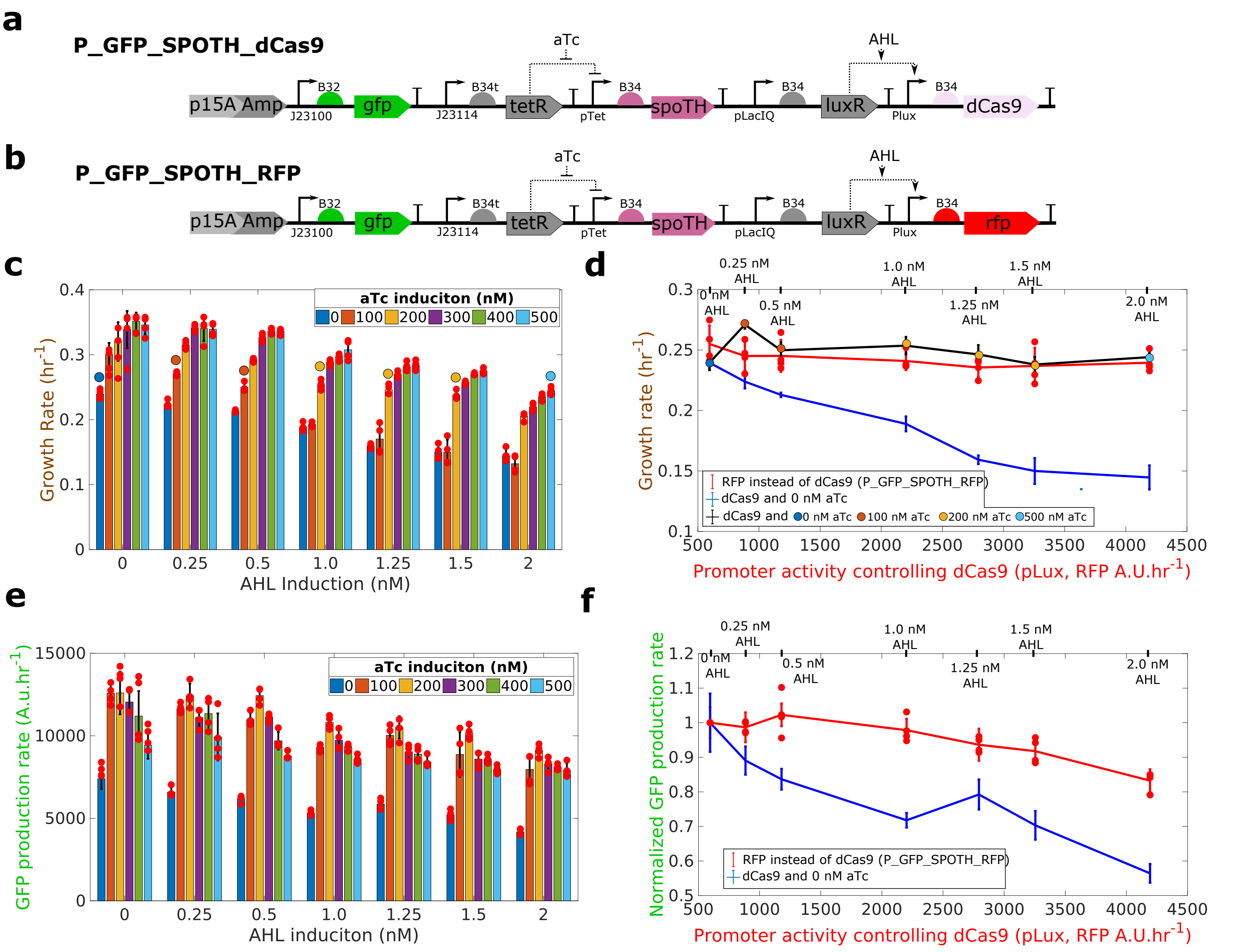

Supplementary Figure. 17: **SpoTH actuator rescues growth rate reduction from dCas9 toxicity. (a)** P_GFP_SpoTH_dCas9 plasmid used to express SpoTH using the inducible pTet promoter and dCas9 using the inducible Plux promoter. **(b)** P_GFP_SpoTH_RFP plasmid used to express SpoTH using the inducible pTet promoter and RFP using the inducible Plux promoter. **(c)** Cell growth rate as dCas9 production rate is increased via AHL, for different levels of SpoTH induction. For each AHL we mark by a circle the aTc level that results in a growth rate closest to the nominal growth rate when AHL = 0 nM and aTc = 0 nM. **(d)** Cell growth rate versus pLux promoter activity as measured in units of RFP production rate for the same AHL induction levels as in (c) when pLux is transcribing RFP (red line, P_GFP_SpoTH_RFP with no SpoTH induction), dCas9 and with no SpoTH induction (blue line), and dCas9 with SpoTH induction corresponding to the colored circles in (c) (black line). **(e)** GFP production rate as dCas9 production rate is increased via AHL, for different levels of SpoTH induction. **(f)** GFP production rate versus pLux promoter activity as measured in units of RFP production rate for the same AHL induction levels as in (e) when pLux is transcribing RFP (red line, P_GFP_SpoTH_RFP with no SpoTH induction) and dCas9 and with no SpoTH induction (blue line). Data are shown as the mean ± one standard deviation (N=4, two biological replicates each with two technical replicates). Individual experimental values are presented as a red dots. All experiments were performed in the CF945 strain in media with glycerol as the sole carbon source. Plasmid description, plasmid map, and essential DNA sequences are provided in Supplementary section *Plasmid maps and DNA sequences*. The complete experimental protocol is provided in the Materials and Methods section.

[13] N. C. Imholz, M. J. Noga, N. J. van den Broek, and G. Bokinsky, "Calibrating the Bacterial Growth Rate Speedometer: A Re-evaluation of the Relationship Between Basal ppGpp, Growth, and RNA Synthesis in Escherichia coli," *Front. Microbiol.*, vol. 11, no. September, pp. 1–9, 2020.

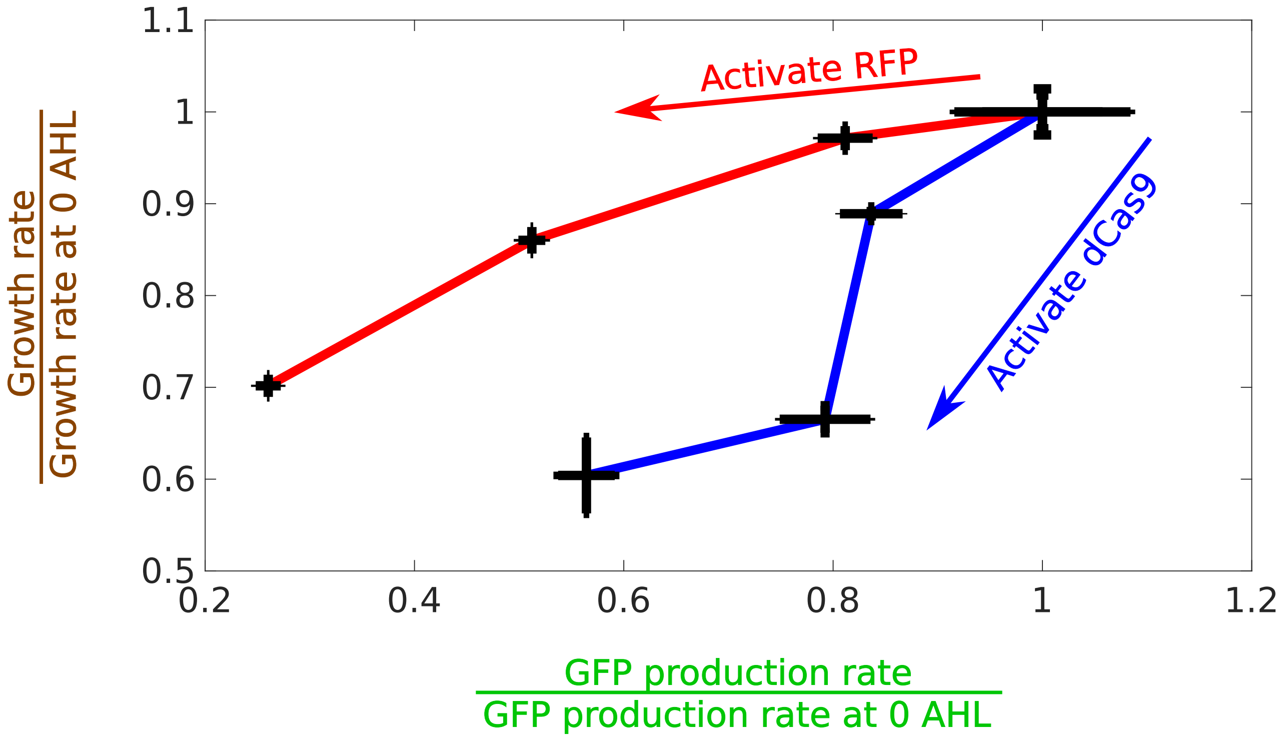

Supplementary Figure. 18: **dCas9 expression places more growth rate burden on the cell than RFP expression due to toxic effects.** dCas9 and RFP are expressed using the P_GFP_SpoTH_dCas9 (blue line) and P_GFP_SpoTH_RFP (red line) plasmids, respectively (Supplementary Fig. 17). The GFP production rate normalized by GFP production rate when there is no AHL induction versus the growth rate normalized by growth rate when there is no AHL induction. The inductions for P_GFP_SpoTH_dCas9 are 0 nM, 0.5 nM, 1.25 nM, and 2 nM AHL and for P_GFP_SpoTH_RFP are 0 nM, 3 nM, 7 nM, 15 nM AHL. Data are shown as the mean ± one standard deviation (N=4, two biological replicates each with two technical replicates). All experiments were performed in the CF945 strain in media with glycerol as the sole carbon source. The complete experimental protocol is provided in the Materials and Methods section.

[14] M. M. Barker, T. Gaal, C. A. Josaitis, and R. L. Gourse, “Mechanism of regulation of transcription initiation by ppGpp. I. Effects of ppGpp on transcription initiation in vivo and in vitro,” *J. Mol. Biol.*, vol. 305, no. 4, pp. 673–688, 2001.

[15] P. P. Dennis, M. Ehrenberg, and H. Bremer, “Control of rRNA synthesis in Escherichia coli: a systems biology approach.,” *Microbiol Mol Biol Rev*, vol. 68, no. 4, pp. 639–668, 2004.

[16] M. Zacharias, H. U. Goringer, and R. Wagner, “Influence of the GCGC discriminator motif introduced into the ribosomal RNA P2- and tac promoter on growth-rate control and stringent sensitivity,” *EMBO J.*, vol. 8, no. 11, pp. 3357–3363, 1989.

[17] P. P. Dennis and H. Bremer, “Modulation of Chemical Composition and Other Parameters of the Cell at Different Exponential Growth Rates,” *EcoSal Plus*, vol. 3, no. 1, 2008.

[18] X. Dai and M. Zhu, “Coupling of Ribosome Synthesis and Translational Capacity with Cell Growth,” *Trends Biochem. Sci.*, vol. 45, pp. 681–692, aug 2020.

[19] I. Shachrai, A. Zaslaver, U. Alon, and E. Dekel, “Cost of Unneeded Proteins in E. coli Is Reduced after Several Generations in Exponential Growth,” *Mol. Cell*, vol. 38, no. 5, pp. 758–767, 2010.

[20] D. Del Vecchio and R. M. Murray, *Biomolecular Feedback Systems.* 2014.

[21] D. R. Gentry and M. Cashel, "Mutational analysis of the Escherichia coli spoT gene identifies distinct but overlapping regions involved in ppGpp synthesis and degradation," *Mol. Microbiol.*, vol. 19, no. 6, pp. 1373–1384, 1996.

[22] H. H. Huang, M. Bellato, Y. Qian, P. Cárdenas, L. Pasotti, P. Magni, and D. Del Vecchio, "dCas9 regulator to neutralize competition in CRISPRi circuits," *Nat. Commun.*, vol. 12, no. 1, pp. 1–7, 2021.

[23] C. D. McBride and D. Del Vecchio, "Predicting Composition of Genetic Circuits with Resource Competition: Demand and Sensitivity," *ACS Synth. Biol.*, vol. 10, pp. 3330–3342, dec 2021.

[24] J. Carrera and M. W. Covert, "Why Build Whole-Cell Models?," *Trends Cell Biol.*, vol. 25, pp. 719–722, dec 2015.

[25] F. Ceroni, R. Algar, G.-B. Stan, and T. Ellis, "Quantifying cellular capacity identifies gene expression designs with reduced burden," *Nat. Methods*, vol. 12, no. 5, pp. 415–418, 2015.

[26] Y. Qian and D. Del Vecchio, "Mitigation of ribosome competition through distributed sRNA feedback," *2016 IEEE 55th Conf. Decis. Control. CDC 2016*, pp. 758–763, 2016.

[27] H. M. Salis, E. A. Mirsky, and C. A. Voigt, "Automated design of synthetic ribosome binding sites to control protein expression," *Nat. Biotechnol.*, 2009.

[28] A. Espah Borujeni, A. S. Channarasappa, and H. M. Salis, "Translation rate is controlled by coupled trade-offs between site accessibility, selective RNA unfolding and sliding at upstream standby sites," *Nucleic Acids Res.*, vol. 42, no. 4, pp. 2646–2659, 2014.

[29] J. M. Rock, F. F. Hopkins, A. Chavez, M. Diallo, M. R. Chase, E. R. Gerrick, J. R. Pritchard, G. M. Church, E. J. Rubin, C. M. Sassetti, D. Schnappinger, and S. M. Fortune, "Programmable transcriptional repression in mycobacteria using an orthogonal CRISPR interference platform," *Nat. Microbiol.*, vol. 2, no. February, pp. 1–9, 2017.

[30] Y. J. Lee, A. Hoynes-O'Connor, M. C. Leong, and T. S. Moon, "Programmable control of bacterial gene expression with the combined CRISPR and antisense RNA system," *Nucleic Acids Res.*, vol. 44, no. 5, pp. 2462–2473, 2016.

[31] P. D. Hsu, D. A. Scott, J. A. Weinstein, F. A. Ran, S. Konermann, V. Agarwala, Y. Li, E. J. Fine, X. Wu, O. Shalem, T. J. Cradick, L. A. Marraffini, G. Bao, and F. Zhang, "DNA targeting specificity of RNA-guided Cas9 nucleases," *Nat. Biotechnol.*, vol. 31, no. 9, pp. 827–832, 2013.

[32] S. H. Sternberg, S. Redding, M. Jinek, E. C. Greene, and J. A. Doudna, "DNA interrogation by the CRISPR RNA-guided endonuclease Cas9," *Nature*, vol. 507, no. 7490, pp. 62–67, 2014.

[33] S. Zhang and C. A. Voigt, "Engineered dCas9 with reduced toxicity in bacteria: Implications for genetic circuit design," *Nucleic Acids Res.*, vol. 46, no. 20, pp. 11115–11125, 2018.
