# Supplementary material for: Feedforward growth rate control mitigates gene activation burden
Source: Nat Commun. 2022 Nov 17;13:7054. doi: 10.1038/s41467-022-34647-1 (PMC9672102; doi:10.1038/s41467-022-34647-1)
Supplement: Supplementary file 4 — Description of Additional Supplementary Files [file 41467_2022_34647_MOESM4_ESM.pdf]

Title: Supplementary Software

Description: The zip file including codes to produce simulation results in Figures 2 and 3.
